# The microbiota metabolite, phloroglucinol, confers long-term protection against inflammation

**DOI:** 10.1080/19490976.2024.2438829

**Published:** 2024-12-15

**Authors:** Janire Castelo, Sarai Araujo-Aris, Diego Barriales, Samuel Tanner Pasco, Iratxe Seoane, Ainize Peña-Cearra, Ainhoa Palacios, Carolina Simó, Virginia Garcia-Cañas, Muthita Khamwong, Itziar Martín-Ruiz, Monika Gonzalez-Lopez, Laura Barcena, José Ezequiel Martín Rodríguez, José Luís Lavín, Naiara Gutiez, Raquel Marcos, Estibaliz Atondo, Arantza Cobela, Laura Plaza-Vinuesa, Adrián Plata, Eneko Santos-Fernandez, Alberto Fernandez-Tejada, Mari Carmen Villarán, José Miguel Mancheño, Juana Maria Flores, Ana María Aransay, Aize Pellón, Blanca de las Rivas, Rosario Muñoz, Abelardo Margolles, Patricia Ruas-Madiedo, Maria Victoria Selma, Mercedes Gomez de Agüero, Leticia Abecia, Juan Anguita, Héctor Rodríguez

**Affiliations:** a CIC bioGUNE, Basque Research and Technology Alliance (BRTA), Derio, Spain; b Department of Immunology, Microbiology and Parasitology, Faculty of Medicine and Nursing, University of the Basque Country (UPV/EHU), Bilbao, Spain; c Molecular Nutrition and Metabolism, Institute of Food Science Research (CIAL), Spanish National Research Council (CSIC), Madrid, Spain; d Würzburg Institute of Systems Immunology, Max-Planck Research Group at the Julius-Maximilians Universität, Würzburg, Germany; e Applied Mathematics Department - Bioinformatics Unit, NEIKER-BRTA, Derio, Spain; f Departamento de Microbiología y Bioquímica, Instituto de Productos Lácteos de Asturias, CSIC, Villaviciosa, Spain; g Functionality and Ecology of Beneficial Microbes (MicroHealth) Group, Instituto de Investigación Sanitaria del Principado de Asturias, Oviedo, Spain; h Departamento de PRocesos Tecnológicos y Biotecnología, Instituto de Ciencia y Tecnología de Alimentos y Nutrición (ICTAN), CSIC, Madrid, Spain; i Ikerbasque, Basque Foundation for Science, Bilbao, Spain; j Área de Alimentación Saludable, Tecnalia-BRTA, Miñano, Spain; k Department of Crystallography and Structural Biology, Institute of Physical Chemistry Blas Cabrera (IQF), CSIC, Madrid, Spain; l Department of Animal Medicine and Surgery, Veterinary Faculty, Universidad Complutense de Madrid, Madrid, Spain; m CIBERehd, Instituto de Salud Carlos III (ISCIII), Madrid, Spain; n Laboratory of Food & Health, Research Group on Quality, Safety and Bioactivity of Plant Foods, CEBAS-CSIC, Murcia, Spain

**Keywords:** Microbiota byproducts, phenolic derivatives, central trained immunity, inflammation

## Abstract

Phloroglucinol is a key byproduct of gut microbial metabolism that has been widely used as a treatment for irritable bowel syndrome. Here, we demonstrate that phloroglucinol tempers macrophage responses to pro-inflammatory pathogens and stimuli. *In vivo*, phloroglucinol administration decreases gut and extraintestinal inflammation in murine models of inflammatory bowel disease and systemic infection. The metabolite induces modest modifications in the microbiota. However, the presence of an active microbiota is required to preserve its anti-inflammatory activity. Remarkably, the protective effect of phloroglucinol lasts partially at least 6 months. Single-cell transcriptomic analysis of bone marrow progenitors demonstrates the capacity of the metabolite to induce long-lasting innate immune training in hematopoietic lineages, at least partially through the participation of the receptor and transcription factor, aryl hydrocarbon receptor (AhR). Phloroglucinol induces alterations in metabolic and epigenetic pathways that are most prevalent in upstream progenitors as hallmarks of central trained immunity. These data identify phloroglucinol as a dietary-derived compound capable of inducing central trained immunity and modulating the response of the host to inflammatory insults.

## Introduction

Gut microbiota can transform a variety of food components, rendering several metabolites that can impact human health. Among dietary substrates, polyphenolic compounds are an abundant component of nutritional sources from vegetal origin. Food phenolics are transformed by the microbiota through cleavage, hydrolysis or reduction steps into compounds with different and frequently increased bioactivities for both the human host and the microbial communities that inhabit the gut.^1^ The absorption of microbiota-derived dietary metabolites by the host has been associated with the modulation of the immune response to infection. For instance, macrophages are more effective against infection when butyrate, a microbial metabolite resulting from the transformation of dietary fiber, is present during differentiation.^2^ On the other hand, symbiont transformation of dietary amino acids is related to the activation of Natural Killer T cells,^3^ while desaminotyrosine (DAT), a metabolite resulting from the transformation of dietary phenolics, improves the outcome of flu virus infection in a mouse model by modulating type I IFN responses in macrophages.^4^ Furthermore, mortality in COVID-19 is associated with reduced levels of this phenolic product resulting from the transformation of vegetables by gut microbes.^5^

An important metabolite in the microbiota transformative pathway of polyphenolic compounds is phloroglucinol. This compound has been proposed as one of the final transformative products after the degradation of different dietary polyphenolic compounds by gut microbes.^6,7^ Among phenolics with a demonstrated role in gut immune modulation in animal models, phloretin,^8^ a component of apples and other vegetables can be transformed to phloroglucinol by gut microbes harboring phloretin hydrolase enzymes. Phloretin inflammatory modulation observed in animal models might be at least in part the result of phloroglucinol production by gut microbes making the study of this compound even more compelling. Phloroglucinol has been detected in the feces of a cohort of individuals after lentil intake,^9^ and some studies point to individual gut microbiota composition as a key determinant for its gut abundance.^10^ Moreover, it attenuates the inflammatory response to LPS in a macrophage-like cell line.^11^ Additionally, it has been used as treatment in Irritable Bowel Syndrome (IBS) patients due to its antispasmodic properties.^12,13^ However, despite its prevalence and previous use of the compound and its putative precursors in the treatment of gut inflammatory conditions, little is known regarding its immune modulatory capacity.

We have explored the role of phloroglucinol as a potent anti-inflammatory compound. *In vitro* and *in vivo* experiments showed that orally administered phloroglucinol improved disease outcomes in intestinal and extraintestinal murine models of inflammatory pathologies defined by exacerbated macrophage responses. Furthermore, we explored the mechanisms underpinning this protection, demonstrating a role for the metabolite as a modulator of both inflammatory responses and microbial composition. Strikingly, phloroglucinol imprinted long-term tolerance in the bone marrow, resulting in lingering resistance to disease. These results underscore the capacity of the host microbiota to affect local and systemic inflammatory responses in response to dietary habits.

## Materials and methods

### Bacterial strains and growth conditions

The bacterial strains used throughout, and their growth conditions are listed in Table S1. Anaerobic bacteria were grown in MG500 (Don Whitley Scientific, West Yorkshire, United Kingdom) and INVIVO2 400 hypoxia station (Ruskinn Technology Ltd, Bridgend, UK) anaerobic chambers. Anaerobic conditions were maintained under an atmosphere of 10% (vol/vol) H_2_, 10% CO_2_ and 80% N_2_.

To calculate the multiplicity of infection (m.o.i.) for each bacterium before stimulation, an estimation of bacterial colony forming units (CFU) in culture was performed. A correlation equation was calculated for each strain, using the association between culture OD at 600 nm and the CFUs determined by dilution plating.

### Ethics statement, mouse strains and husbandry

C57BL/6J mice were purchased from Charles River laboratories (Lyon, France) and maintained under specific pathogen-free conditions with controlled temperature (22 ± 1 °C) and 12/12-hour light/dark cycles. Mice were fed ad libitum on standard mouse chow (irradiated Teklad Global diet 2914, Envigo, Horst, Netherlands, https://insights.inotiv.com/hubfs/resources/data-sheets/2914.pdf).

Animal protocols were approved by the Animal Research Ethics Board of CICbioGUNE and the Competent Authority (Diputación de Bizkaia) according to the guidelines of the European Union Council (Directive 2010/63/EU) and Spanish Government regulations (RD 53/2013). The Animal Facility at CICbioGUNE is accredited by AAALAC Intl.

Germ-free C57BL/6J mice were bred and maintained in flexible film isolators at the University of Würzburg (Institute of Systems Immunology) as described by Smith et al.^14^ The mice were provided autoclaved (132°C for 20 min) diet 3307 from Kliba-Nafag (htpps://www.kliba-nafag.com/en/products/standard-diets/3307-special-strains-vitamin-fortified/). Germ-free status was routinely monitored by bacterial aerobic and anaerobic cultures and bacterial DNA labeling imagery, and a screen for parasites, bacteria and virus was performed every three months. C57BL/6J wild type and *Ahr*-deficient mice (B6;129-Ahrtm1Bra/J);^15^ were housed in individually ventilated cages at the University of Würzburg (Institute of Systems Immunology). SPF mice were regularly tested for pathogens and fulfilled the requirements for pathogens of the Federation of European Laboratory Animal Science Associations (FELASA). All mouse experiments were performed in accordance with the institutional guidelines of the Lower Franconia Government.

### Growth kinetics of bacteria in the presence of phloroglucinol

*F. nucleatum, E. coli, A. muciniphila* and *P. copri* growth was evaluated at increasing concentrations of phloroglucinol (Sigma-Aldrich, Merck Life Science, Madrid, Spain; 5, 2.5 and 1.25 mM) using a 96-well plate. 2.5 µl of the preculture (1:100 dilution) were loaded into a final volume of 250 µl and the plate was incubated overnight under anaerobic conditions at 37°C. The following morning, the optical density was recorded at 600 nm at 2 h intervals for 12 h and at 24 h. Inoculated and uninoculated wells of phenolic-free broth, as well as uninoculated wells with phloroglucinol were used as controls. Two independent experiments were performed with 9 replicates each.

### Murine bone marrow-derived macrophages (BMMs)

Bone marrow cells were isolated from tibias and femurs and differentiated as previously described.^16^ Briefly, bone marrow cells were subjected to erythrocyte lysis and incubated in non-treated dishes (Fisher Scientific, Alcobendas, Spain), at 37°C in complete DMEM (Lonza, Basel, Switzerland) supplemented with 30 ng/ml of M-CSF (Miltenyi Biotech, Pozuelo de Alarcón, Spain). The cytokine was refreshed on day 3, and, on day 6, the differentiated macrophages were scraped, counted, and seeded at a concentration of 1 × 10^6^ cells/ml.

### In vitro determination of metabolites cell toxicity

BMMs were seeded into 24-well plates at a density of 2.5 × 105 cells/well and incubated overnight. The cells were then incubated in 500 µl of DMEM with 10% FBS (Fisher Scientific) containing phloroglucinol, phloretic acid, methyl gallate, gallic acid, pyrogallol or phloretin at 0, 0.01, 0.1 and 1 mM for 20 h. A control condition without compound but with solvent (EtOH 96%) was included. Subsequently, the cells were washed with PBS containing 1% FBS and incubated in 500 µl of DMEM supplemented with 10% FBS, containing 44 µM resazurin (Stem Cell Technologies, Saint Égrève, France) for 3 h. The fluorescence (Ex 530/Em 590 nm) of 100 µl of the supernatant was measured in a flat-bottom 96 well black plate (Corning, New York, USA) on a plate reader (Biotek Instruments, Winooski, USA).

### In vitro stimulation of macrophages

BMMs were seeded at a density of 5 × 10^5^ cells/well into 12-well plates and left to attach. To study the direct effect of phloroglucinol and phloretic acid (Sigma-Aldrich) in macrophages, the compounds (0, 0.01, 0.1 and 1 mM) were added to the cells and incubated at 37°C for 1 h prior to the addition of the stimuli. The cells were stimulated for 20 h with 10 ng/ml lipopolysaccharide (LPS) from *Salmonella enterica* (Sigma-Aldrich) and with two live proinflammatory pathogens, *F. nucleatum* and a clinical isolate of *E. coli* at an m.o.i. of 1 or left unstimulated. The supernatants were then collected and centrifuged at 9,300 ×g for 10 min and stored at −80°C until analyzed by ELISA.

To study the long-term effect of the metabolites in macrophages, the compound was added at the indicated concentrations and the cells were incubated for 20 h at 37°C, washed, and rested for 24 h prior to the addition of the stimuli in the absence of the metabolite.

Phloroglucinol-treated mice were sacrificed directly after phloroglucinol treatment or after a resting time without phloroglucinol. BMMs were collected and processed as described above. Then, BMMs were stimulated with an m.o.i of 10 for *F. nucleatum*, a clinical isolate of *E. coli* and *E. coli Nissle 1917* and with an m.o.i of 25 for *B. burgdorferi* or were left unstimulated.

### *In vitro stimulations with phloroglucinol and* B. burgdorferi

For *in vitro* acute-single stimulations, 5 × 10^5^ BMMs were seeded in DMEM with 10% FBS and 1% P/S onto 24-well plates (Fisher Scientific) and incubated at 37°C and 5% CO_2_. After 3 h, cells were treated with 1 mM phloroglucinol for 1 h before stimulating them overnight with *B. burgdorferi* B31 at an m.o.i. of 25.^16^ Supernatants were collected the following day and stored at −80°C until analyzed by ELISA.

*B. burgdorferi*-induced training assays were performed as described.^16^ Trained macrophages were pretreated with 1 mM phloroglucinol 1 h before the second stimulation with the spirochete.

### Cytokine ELISA

The levels of murine IL-6 and TNF in the supernatants were measured by ELISA using the Mouse IL-6 ELISA Set and Mouse TNF ELISA Set II (BD Biosciences), following the manufacturers’ instructions.

### Treatment of mice with phloroglucinol

To study the anti-inflammatory capacity of phloroglucinol *in vivo*, the compound was administered at a concentration of 1 mg/ml in the drinking water to 6–12-week-old mice. The mice were treated for 14 days, unless otherwise stated, followed by the indicated periods of resting in the absence of phloroglucinol. All experimental groups were compared to non-treated mice as control groups.

### Phloroglucinol quantification in fecal, urine and serum samples

The concentration of phloroglucinol in feces, urine and serum was determined by an UPLC-MS/MS method adapted from Li et al.^17^ Phloroglucinol was extracted from fecal samples by mixing 40 mg of feces with 50 µl 1% HCl, 20 µl of 1 µg/ml acetaminophen and 800 µl ethyl acetate (Sigma-Aldrich). For urine and serum samples, phloroglucinol was extracted by adding 25 µl 1% HCl, 10 µl of 1 µg/ml acetaminophen and 400 µl ethyl acetate (Sigma-Aldrich) to 50 μl of sample. Mixtures were then homogenized in a FastPrep-24 5 G (MP Biomedicals, Irvine, CA). Homogenates were vortex-mixed at 1,000 rpm for 15 min at 10°C in a Thermomixer C (Eppendorf, Hamburg, Germany), and then centrifuged at 4,000 ×g and 10°C for 15 min. For each sample, 300 µl of the upper organic phase was transferred to a new vial and evaporated under a stream of nitrogen. The residue was reconstituted in 50 µl of LC-MS grade acetonitrile (ACN, VWR, Radnor, PA)-water (10:90, v/v) by vortex-mixing for 10 min at 10°C in a Thermomixer C. After being centrifuged at 14,000 ×g and 10°C for 10 min, the supernatants were transferred to an LC vial and analyzed by UPLC-MS/MS. To quantify phloroglucinol, a calibration curve at a working range of 0.001–4 µg/ml was prepared and analyzed by UPLC-MS/MS. Analyses were performed on an Agilent 1260 Infinity II and Ultivo 6465 Triple Quadrupole LC/MS system, equipped with Jet Stream ESI source from Agilent Technologies (Santa Clara, CA, USA). Phloroglucinol and acetaminophen were separated on a ZORBAX StableBond C8 (2.1 × 100 mM, 1.8 µm) column maintained at 40°C. Samples were injected into LC using an auto-sampler (1 µl, maintained at 8 °C). Mobile phases consisted of 0.01% formic acid in water (A) and 0.01% formic acid in ACN (B), at a total flow rate of 0.4 ml/min with the following gradient: 0 min, 5% B; 1.5 min, 5% B; 3 min, 100% B; 5 min, 100% B, and thereafter back to the initial condition (5% B) and equilibrated for 3 min. The ion spray voltage in negative mode was set at 4.0 kV, gas temperature and flow rate were 300°C and 10 ml/min, respectively. Nebulizer pressure was 40 psi, sheath gas temperature was set at 350°C, and sheath gas flow was 12 ml/min. After optimization, MRM detection mode was performed with the transitions at m/z 125.0 → 56.9 for phloroglucinol and 150.1 →106.9 for IS.

### Dextran sulfate sodium (DSS)-induced acute colitis mouse model

Acute colitis was induced in 6–12-week-old mice with 3% DSS (TdB Labs, Uppsala, Sweden) in the drinking-water for 7 days, followed by a resting period of 2 days. Animal body weight, the presence of gross blood in feces, and stool consistency were individually evaluated. Each parameter was assigned a score according to the criteria proposed previously and used to calculate an average daily disease activity index (DAI).^18^ The last day of treatment with phloroglucinol feces were collected. The day of sacrifice colon length, spleen weight, colon content, colon samples and sera were collected.

Colon tissues were fixed in 10% formalin, dehydrated, embedded in paraffin and cut into 5 μm-thick sections. For histopathology, sections were deparaffined, hydrated and stained with hematoxylin and eosin. Stained sections were analyzed and blindly given a score by a pathologist. The histological scores were based on those described elsewhere.^18^

### Myeloperoxidase activity assay

One centimeter of the distal colon was homogenized in 50 mM phosphate buffer (pH 6.0) and 0.5% hexadecyltrimethylammonium bromide (CTAB, Sigma-Aldrich) using a Precellys 24 homogenizer (Bertin Technologies, Montigny-le-Bretonneux, France). Myeloperoxidase (MPO) activity was then determined as described.^19^

### *Murine infection with* B. burgdorferi

Mice were infected with 10^5^ bacteria subcutaneously in the interscapular area, using a solution of 10^6^ spirochetes/ml in PBS, as described.^16^ At sacrifice, blood was drawn by intracardiac puncture, and the sera was used to confirm infection by Western blot. Additionally, the hearts were cut in half with the aorta as reference and through the ventricles. DNA and RNA were isolated following the manufacturer’s instructions from the AllPrep DNA/RNA/miRNA Universal Kit (Qiagen, Las Rozas, Spain).

Heart DNA was used to measure bacterial burdens by quantitative polymerase chain reaction (qPCR) targeting *recA* expression relative to the murine gene, *Rpl19*. Heart RNA was reverse-transcribed with M-MLV (Thermo Fisher Scientific). The expression levels of *Tnf* and *Adgre1* relative to *Rpl19* were then determined by real-time PCR. PCRs were performed using the PerfeCTa SYBR Green SuperMix low ROX (Quantabio, Beverly, Massachusetts, USA) on a QuantStudio 6 Real-Time PCR System (Thermo Fisher Scientific). PCR efficiency was always between 90 and 110%. The primers used are listed in Table S2.

### Fecal DNA extraction

DNA was isolated from frozen stool samples (40 mg) using the FavorPrep Stool DNA Isolation Mini kit (Favorgen, Vienna, Austria) following the manufacturer’s instructions with the lysis temperature increased to 95°C. Samples were eluted with 50 microliters µl of nuclease-free Hyclone water (Thermo Fisher Scientific) and assessed spectrophotometrically with a NanoDrop ND-100 Spectrophotometer (Thermo Fisher Scientific). Purified DNA samples were stored at −20°C until used.

### Microbiome analysis

Phylogenetic-based methods targeting the 16S rRNA gene were used to characterize the microbial populations present in the feces of experimental mice. DNA extracts were used as the template for PCR-based amplification of the bacterial V3-V4 region of the 16S rRNA, and Illumina adapters were added by a second PCR (indexing PCR). The resulting libraries were then sequenced in an Illumina Miseq platform using 300 bp paired-end reads to obtain a minimum of 60,000 reads per library.

Data processing was performed using mothur (v.1.44.1)^20,21^ following the MiSeq standard operating procedure.^22^ Briefly, sequencing files were combined using the make.contigs command, followed by unique.seqs and their alignment to the Silva database (version 138.2)^23^ (align.seqs). Base ambiguity and long-read sequences were filtered using screen.seqs and filter.seqs, followed by unique.seqs in order to remove redundancy. The sequences were then pre-clustered and devoid of chimeras using the chimera.vsearch command. The sequences were then classified (classify.seqs) and filtered with remove.lineage, using taxon=Chloroplast-Mitochondria-unknown-Archaea-Eukaryota.

Annotation of the sequences was performed using the commands cluster.split with a cutoff value of 0.03, dist.seqs, sens.spec and make.shared, followed by classify.otu. Finally, a phylogenetic tree was generated using get.oturep, dist.seqs and clearcut.

Downstream analysis was performed in R (v. 4.2.2) using RStudio (v. 2023.06.0 + 421) and the Phyloseq package,^24^ as described in https://norwegianveterinaryinstitute.github.io/BioinfTraining/phyloseq_tutorial.html, with some modifications. Normalization was performed by rarefication as suggested. The identification of significant fold changes of OTUs was performed with DESeq2.^25^

### Determination of fecal levels of SCFAs

Concentrations of SCFAs (acetate, propionate and butyrate) in murine feces were determined by an UPLC-MS/MS method adapted from Han et al.^26^ Fecal samples (50 mg) were disrupted and homogenized in 500 μl ACN-water (50:50, v/v) containing 5 µM 2-methyl-valeric acid (Sigma-Aldrich) in a FastPrep-24 5 G homogenizer. Homogenates were vortex-mixed at 1,000 rpm and 10°C for 15 min in a Thermomixer C, and then centrifuged at 14,000 ×g and 4°C for 15 min. Supernatants were collected and derivatized prior to UPLC-MS/MS analysis. To quantify SCFAs, a calibration curve at working range of 1–10000 µM for acetate and 0.1–1000 µM for propionate and butyrate (Sigma-Aldrich) was prepared before SCFAs derivatization. Aliquots (40 μl) of the fecal extracts, calibration standards and extraction blanks were derivatized with 20 μl of 200 mM 3NPH in ACN-water (50:50, v/v) and 20 μl of 120 mM *N*-(3-dimethylaminopropyl)-N’-ethylcarbodiimide hydrochloride (EDC, Sigma-Aldrich)) with 6% pyridine (Sigma-Aldrich) in ACN-water (50:50, v/v). Samples were incubated at 40°C in a Thermomixer C for 30 min. After derivatization, the mixtures were 20-fold diluted with 1,520 μl ACN-water (10:90, v/v). The derivatized samples were run through an integrated instrument composed of an Agilent 1260 Infinity II and Ultivo 6465 Triple Quadrupole LC/MS system, equipped with Jet Stream ESI source from Agilent Technologies. Chromatography separation was performed on an Agilent ZOBAX Eclipse Plus C18 (2.1 × 100 mM, 1.8 µm) maintained at 40°C. Samples were injected into LC using an auto-sampler (1 µl, maintained at 8 °C). Mobile phases were 0.01% formic acid in water (A) and 0.01% formic acid in ACN (B) and it was delivered at a flow rate of 0.65 mL/min with the following gradient: 0 min, 5% B; 5 min, 20% B; 9 min, 50% B; 10 min, 100% B; 12 min, 100% B, and thereafter back to initial condition (5% B) and equilibrated for 3 min. Multiple Reaction Monitoring (MRM) acquisition conditions were set to provide the best sensitivity and specificity for each analyte while the mass transition ion-pair was followed as m/z 194 → 137 for acetate, 208.1 →137 for propionate and 222.1 → 137 for butyrate. The source and gas parameters for the mass spectrometer were set as follows: ion spray voltage 4.0 kV, gas temperature: 300°C, gas flow 10 ml/min, nebulizer pressure 40 psi, sheath gas temperature: 350°C, sheath gas flow: 12 ml/min. An example chromatogram is shown in Figure S1.

### Transcriptional analysis of bone marrow progenitors (scRNAseq)

After bone marrow isolation and red cell lysis, lineage negative (Lin–) cells were enriched using magnetically-activated cell separation (MACS) columns (Miltenyi Biotec) following incubation with a biotinylated lineage antibody cocktail (Miltenyi Biotec) and anti-biotin magnetic beads (Miltenyi Biotec). Enriched Lin – cells were stained with the biotinylated lineage cocktail, cKit (Miltenyi Biotec), anti-biotin (Miltenyi Biotec), and annexin V (Miltenyi Biotec). Prior to sorting, cells were stained with DAPI to check their viability. Cells were sorted using a BD FACSFusion, and Lin – cKit+ Annexin V – DAPI – cells from each mouse were captured in PBS with 0.04% BSA. Equal numbers of sorted cells from each mouse were combined into pools for each experimental group, and each group sample was loaded onto a 10X Genomics chip (10X Genomics, Leiden, The Netherlands).

Single cell libraries targeting 10,000 cells per sample were prepared using the “Chromium Next GEM Chip G Single Cell Kit”, “Chromium Next GEM Single Cell 3ʹ Kit v3.1”, and “Dual Index Kit TT Set A”, following the manufacturer’s user guide (v3.1). Briefly, after loading 20,000 cells in the chip, a pool of ~ 3,500,000 10× barcodes was sampled separately to index each cell’s transcriptome by partitioning the loaded cells into nanoliter-scale Gel Beads-in-emulsion (GEMs), so that all cDNAs generated per cell shared a common 10× barcode. Then, dual indexed libraries were generated from 25–150 ng of amplified, purified and quantified cDNA. Final libraries were quantified using the Qubit dsDNA HS DNA kit (Thermo Fisher Scientific) and visualized on an Agilent 2100 Bioanalyzer using the Agilent High Sensitivity DNA kit (Agilent Technologies) and quantified using Qubit dsDNA HS DNA Kit (Thermo Fisher Scientific). The libraries were then sequenced on a NovaSeq 6000 instrument (Illumina Inc.) to obtain a minimum of 20,000 paired-ended reads per targeted cell.

Raw reads were aligned to the mouse mM10 reference genome, the counts separated by cell and the data filtered and processed using Cell Ranger version 6.1.2 according to manufacturer recommendations.^27^

For statistical analysis of the scRNAseq data set, processed 10X files were uploaded into RStudio (version 2023.06.0 + 421) and processed with Seurat (version 4.3.0),^28^ following default, standard, or generally recommended settings. Quality control was performed using filtration criteria to allow for cells containing at least 1,000 genes per cell and fewer than 10% mitochondrial genes for analysis. Predicted doublets were removed using DoubletFinder.^29^ To account for variations due to cell cycle effects, cell cycle scaling was applied by predicting cell cycle phase using canonical markers^30^ and regressing out the difference between assigned G2M and S phase scores, as described in the Seurat manual for murine hematopoiesis.

To control for batch effects, integration of both datasets was performed by defining 3,000 anchors (FindIntegrationAnchors function) prior to merging (IntegrateData function). Principal component (PC) analysis (PCA) was performed (RunPCA function), and the first 50 PCs were selected to generate a uniform manifold approximation and projection (UMAP) (RunUMAP function). Cells were clustered using the FindNeighbors function (reduction = “pca”, dims = 1:50) then FindClusters (resolution 0.7), resulting in 11 clusters.

After using the FindAllMarkers to identify unique markers per cluster, annotation of cluster identity was assigned based on expression of defined hematopoietic markers^31^ and cell cycle genes.^30^ Differential expression analysis was performed with the FindMarkers function, and genes with FDR < 0.05 were considered significant. Lists of up- and down-regulated DEGs were uploaded into the HOMER software^32^ to perform transcription factor motif enrichment analysis and gene ontology analysis.

### Ethoxyresorufin-O-deethylase (EROD) assay

AhR activation was measured by determining the activity of the AhR-induced enzyme, CYP1A1,^33^ as described.^34^ Briefly, colonic tissue samples were cut open and washed for 20 min at 37°C with 200 r.p.m. shaking in DMEM 1% FBS, 10 mM HEPES, 1% penicillin/streptomycin, and 2 mM DTT. Colon samples were then cut into small pieces and digested in DMEM 1% FBS, 10 mM HEPES, 1% penicillin/streptomycin, 50 μg/ml DNase I (Roche), and 0.4 mg/ml Liberase TL (Roche) for 30 min at 37°C with 200 r.p.m. shaking. Cell suspensions were then filtered through 70-µ cell strainers, pelleted and resuspended in sodium phosphate buffer (PB; 50 mM, pH 8.0). For the determination of CYP1A1 activity in BMMs, the cells were scrapped after stimulation, pelleted and resuspended in PB. For the EROD assay, the cells were incubated with 2 μM 7-ethoxyresorufin in PB at 37°C for 30 min.^34^ To finish the reaction, and measure protein concentration for normalization purposes, 150 µg/ml of fluorescamine in acetonitrile were added. Resorufin (excitation/emission 535/590 nm) and fluorescamine (excitation/emission 390/485 nm) fluorescence was quantified, and the concentration of each compound was determined using standard curves for resorufin and bovine serum albumin, respectively.

### Data and materials availability

Raw sequences from the metagenomic analysis have been deposited in the European Nucleotide Archive (ENA) under the project number PRJEB67518. The scRNAseq data have been deposited at GEO with the identification, GSE246268. All data are available in the main text of the supplementary materials.

### Quantification and statistical analysis

The results are shown as the means ± standard error (SE). The student’s t test was used to calculate significant differences between means and multiple comparisons were analyzed by ANOVA, followed by pairwise comparisons. A *p* value < 0.05 was considered significant. Statistical calculations were performed with GraphPad Prism version 10.

## Results

### Phloroglucinol modulates pro-inflammatory macrophage responses to IBD related pathobionts

**Figure 1.**
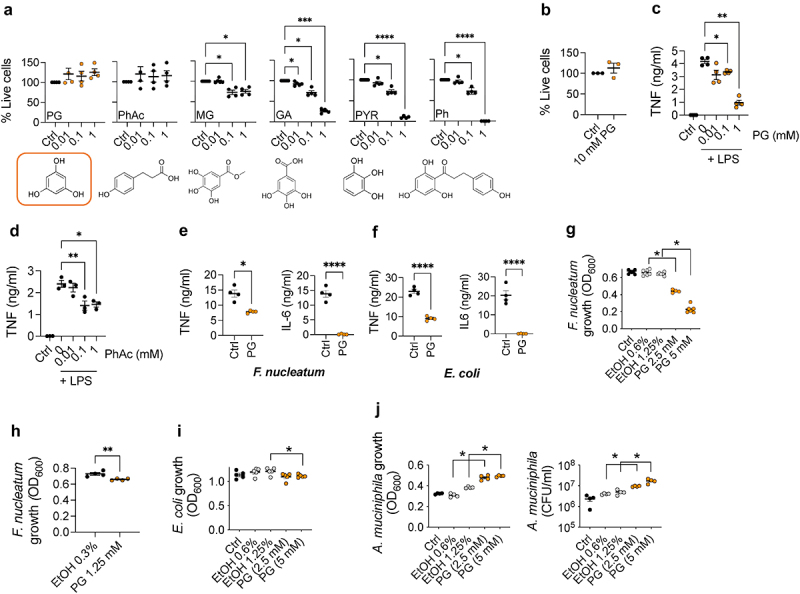
Phloroglucinol modulates pro-inflammatory macrophage responses to Crohn´s related pathobionts. (a) Effect of increasing concentrations (0, 0.01, 0.1 and 1 mM) of phloroglucinol and a panel of chemically related compounds (phloretic acid -PhAc, methyl gallate -MG, gallic acid -GA, pyrogallol -PYR and phloretin -Ph) on BMM cell viability. The chemical structure is represented below the graphs. (b) Effect of 10 mM phloroglucinol (PG) on BMM viability. TNF production in response to LPS in the presence of different concentrations of phloroglucinol (orange, c) and phloretic acid (d). TNF and IL-6 production by BMMs exposed to 1 mM phloroglucinol and stimulated with *F. nucleatum* (e) and *E. coli* (f). Unexposed controls (black) and phloroglucinol (PG) (orange). The figures constitute a representative experiment with the results from 3–4 independent mice. Maximal growth capacity, measured as absorbance at 600 nm after 48 h of *F. nucleatum* (g, h)) and 24 h of *E. coli* (i) in the absence (control; black) or presence of phloroglucinol (orange) or EtOH controls (grey). (j) Maximal growth capacity measured as absorbance at 600 nm and CFU/ml after 48 h of *A. muciniphila* growth in the presence (orange) or absence (black) of phloroglucinol or EtOH controls (grey). *, *p* < 0.05, **, *p* < 0.01, ***, *p* < 0.001, ****. *p* < 0.0001; one-way ANOVA.

We first tested the viability of bone-marrow-derived macrophages (BMMs) when exposed to increasing concentrations of phloroglucinol and a panel of structurally related compounds, such as phloretic acid, methyl gallate, gallic acid, pyrogallol and phloretin. Except for phloroglucinol and phloretic acid, the exposure of BMMs to the compounds resulted in their decreased viability that was generally noticeable at 0.1 mM (Figure 1(a)). In contrast, BMMs exposed to phloroglucinol, including a high dose of 10 mM (Figure 1(b)) did not show signs of decreased viability. We next determined the anti-inflammatory potential of phloroglucinol, by exposing BMMs to a combined stimulation of the compound and different pro-inflammatory insults, including lipopolysaccharide (LPS) and two live pathobionts related to gut pro-inflammatory responses, *Fusobacterium nucleatum* and a clinical isolate of *Escherichia coli*. As a control, we also tested the effect of phloretic acid, due to its lack of toxicity toward BMMs. In response to LPS, tumor necrosis factor (TNF) production was significantly attenuated by phloroglucinol in response to LPS stimulation (Figure 1(c)), while the effect observed in the presence of phloretic acid was less pronounced, particularly at 1 mM concentration (Figure 1(d)). Phloroglucinol also prevented the induction of both TNF and IL-6 in response to *F. nucleatum* (Figure 1(e)) and *E. coli* (Figure 1(f)), suggestive of its anti-inflammatory potential.

To determine whether the repression of proinflammatory cytokine induction by *F. nucleatum* and *E. coli* was dependent on its ability to affect bacterial viability, we assessed the antimicrobial activity of phloroglucinol against both microorganisms. The effect of phloroglucinol on *F. nucleatum* growth was inhibitory at doses ranging from 2.5 to 5 mM (Figure 1(g)), but much less pronounced at a concentration of 1.25 mM (Figure 1(h)). Moreover, phloroglucinol did not affect *E*. coli growth at 2.5 mM and induced a slight decrease in bacterial growth at 5 mM (Figure 1(i)), indicating that the anti-inflammatory effect exerted by the compound upon stimulation of BMMs with both bacteria at the highest concentration tested (1 mM) was not due to its antibacterial effect. Interestingly, and in contrast with the inhibitory effect of phloroglucinol on *F. nucleatum* and *E. coli*, we observed that the presence of the phenolic compound induced a dose-dependent increase in *Akkermansia muciniphila* growth (Figure 1(j)). Overall, these results showed that phloroglucinol is a strong anti-inflammatory metabolite.

### Oral intake of phloroglucinol induces a protective effect in models of acute intestinal and extraintestinal inflammatory diseases

**Figure 2.**
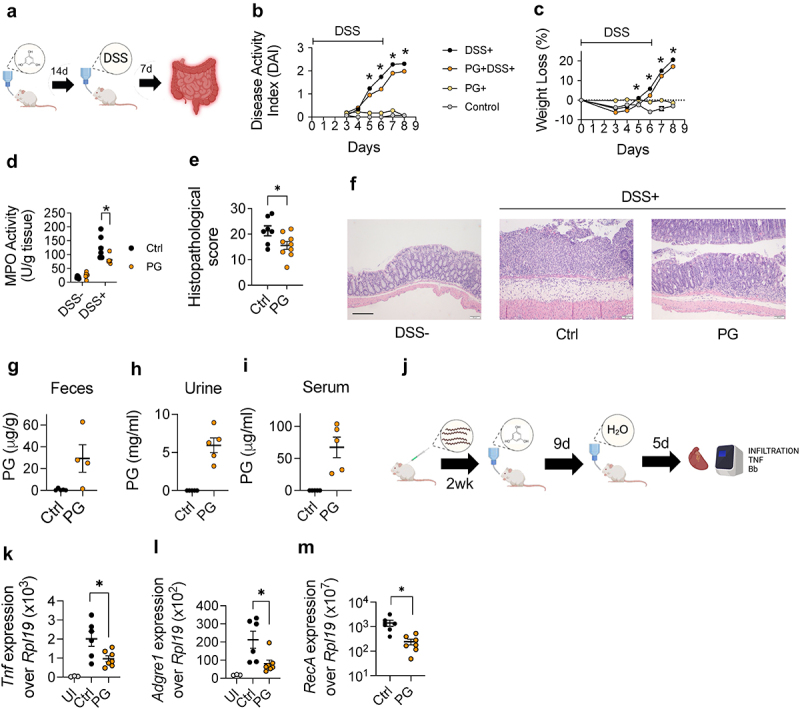
Oral intake of phloroglucinol induces a protective effect in intestinal and extraintestinal inflammatory models. (a) Experimental design: mice were administered phloroglucinol for 14 days in the drinking water, then 3% DSS in water for 6 days, followed by 2 days of no supplemented water. DAI (b) and weight loss (c) over the experimental period in dss-treated mice previously administered phloroglucinol (orange) or vehicle (black) and controls without DSS administration previously treated with phloroglucinol (light yellow) or vehicle (light grey); data are expressed as means  ±  SEM (*n* = 6); *p < 0.05 versus DSS control group. (d) MPO activity in mice treated with phloroglucinol (orange) or vehicle (black). Histopathological scores (e) and representative micrographs (f) of colonic tissue in mice treated with phloroglucinol (orange) and controls (black). Phloroglucinol detection in feces (g), urine (h) and serum (i) of treated (orange) and control mice (black). (j) Experimental design for the experiments testing the effect of phloroglucinol in the murine model of Lyme borreliosis. *Tnf* (K), *Adgre1* (l) and *B. burgdorferi RecA* (m) gene expression relative to *Rpl19* in the heart of phloroglucinol- (orange) and control-treated (black), infected mice, determined by real-time PCR. UI: uninfected mice. The data shown are representative of at least 2 independent experiments.

We then assessed the potential anti-inflammatory properties of phloroglucinol in dextran sulfate sodium (DSS)-induced colitis in mice treated with the compound for two weeks before experimental colitis induction (Figure 2(a)). Phloroglucinol intake attenuated DSS-induced disease activity index (DAI; Figure 2(b)) and weight loss (Figure 2(c)) from day 5 onward, as well as myeloperoxidase (MPO) activity levels at the end of the experiment (Figure 2(d)). The analysis of the histopathological scores also showed a significant reduction in phloroglucinol-treated mice (Figure 2(e,f))

We next investigated the presence of phloroglucinol in fecal samples from mice after oral intake. At the end of the treatment period, the compound was readily detected in the feces of the treated mice (Figure 2(g)). Phloroglucinol was also detected in urine samples at the mg/ml range (Figure 2(h)), indicating that the compound was absorbed and likely eliminated through kidney filtration. Moreover, phloroglucinol was detected in mouse sera (Figure 2(i)). These results indicated that phloroglucinol is absorbed in the intestine and excreted through the urine, while remaining at low levels in the bloodstream.

The absorption capacity of phloroglucinol suggested that the oral treatment with the phenyl derivative may distribute to extraintestinal sites and attenuate inflammatory responses. We, therefore, studied a potential immune modulatory effect of phloroglucinol during Lyme carditis, an inflammatory condition caused by the response of macrophages to the presence of *Borrelia burgdorferi* in the heart^16^ while having no pathological effects on the intestinal tract. Groups of mice were infected with the spirochete. At the peak of the disease (2 weeks of infection), mice were treated with phloroglucinol in the drinking water for a period of 9 days and analyzed after a 5-day period of resting without phloroglucinol (Figure 2(j)). The treatment resulted in the reduction of *Tnf* expression (Figure 2(k)) and macrophage infiltration (measured by the expression of the Adhesion G protein-coupled receptor E1 gene (*Adgre1*)^35^) in the cardiac tissue (Figure 2(l)). Moreover, the treatment with phloroglucinol resulted in a significant reduction of spirochetal levels in the heart (Figure 2(m)). We discarded a direct antimicrobial effect of the compound, since phloroglucinol did not affect spirochetal growth in culture except at very high concentrations (Figure S2a). We also tested the effect of phloroglucinol on the phagocytic activity of BMMs. The incubation with the compound did not affect the ability of macrophages to internalize *B. burgdorferi* (Figure S2b,c). These results showed that orally administered phloroglucinol reaches the bloodstream and modulates systemic macrophage-mediated inflammation.

### Phloroglucinol modifies minimally microbiota composition

**Figure 3.**
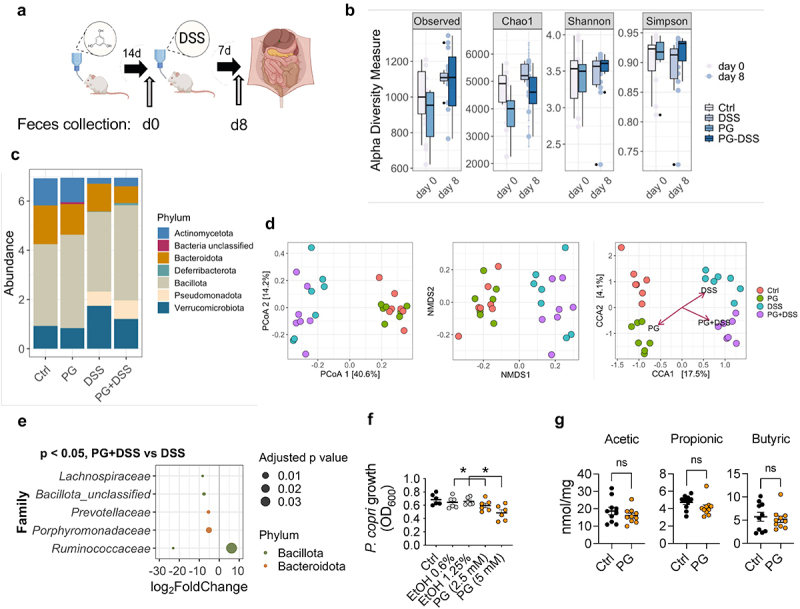
Phloroglucinol minimally modifies microbiota composition. (a) Experimental design: mice received phloroglucinol for 14 days, then 3% DSS for 6 days, followed by 2 days of regular drinking water. Fecal samples were taken for microbiota analysis at the end of the treatment and after DSS administration. (b) Alpha diversity indices for fecal microbiomes across different experimental groups. (c) Microbiota composition at the phylum level. (d) Beta diversity plots: PCoA, NMDS and CCA. (e) Operational taxonomic units significantly different (q < 0.05 FDR) between phloroglucinol-treated and untreated mice after DSS administration. The right side represents OTU’s with a log_2_ fold positive difference for mice treated with phloroglucinol versus untreated mice while the right side is the negative fold change. Each point represents a single OTU colored by phylum and grouped by taxonomic family level, while the size of the points reflects the adjusted *p* value. (f) Maximum growth capacity measured as absorbance at 600 nm of *P. copri* growth in the presence (orange) and absence (black) of phloroglucinol or EtOH controls (grey). (g) Fecal SCFAs levels in phloroglucinol-treated and control mice, analyzed at the end of the treatment (14 d).

Microbial metabolites prevent DSS-induced damage in mice when applied before induction of the disease (Figure 2(a-d)),^36^ and a preexistent microbiota might determine the severity of inflammatory episodes.^37^ To assess whether the protection elicited by phloroglucinol was due to changes in the gut microbial population, the fecal microbiota composition of phloroglucinol-treated mice and controls was analyzed at two different time points, after 14 days of phloroglucinol intake (d0: Ctrl and PG) and after the induction of colitis by DSS administration (d8: PG and PG+DSS; Figure 3(a)). Alpha diversity indexes (Observed, Chao1, Shannon and Simpson) showed no significant differences between phloroglucinol-treated and untreated groups (Figure 3(b)). We then analyzed the relative abundance of key taxa related to gastrointestinal inflammatory diseases.^38^ At the phylum level, the treatment with DSS induced the most important changes, including the increase in Verrucomicrobiota and the reduction in the levels of Actinomycetota (Figure 3(c), Figure S3). On the other hand, the previous treatment with phloroglucinol resulted in increased abundance of Bacillota at day 8 (PG-DSS compared to the DSS group, Figs. 3c and S3). Accordingly, multivariate methods searching for community dissimilarities (PCoA and NMDS1) showed differences between DSS-treated and untreated mice but not between mice treated with phloroglucinol and their respective untreated groups. Only when a constrained analysis was applied, dissimilarities between phloroglucinol-treated and untreated mice were found at both time points (Figure 3(d)). Moreover, at the family level, 5 operating taxonomic units (OTUs) belonging to the Bacteroidota and Bacillota phyla were significantly decreased in phloroglucinol-treated versus control, DSS-induced mice (Figure 3(e)). Among Bacteroidota, the presence of the *Prevotella* genus is especially relevant in terms of inflammation control.^37,39^ The analysis of the antimicrobial effect of phloroglucinol over *P. copri* in culture showed a significant reduction when the microorganism was cultured in the presence of the phenolic metabolite (Figure 3(f)). Overall, these results showed that the protective effect of phloroglucinol on DSS-induced inflammation may occur in part by direct and indirect effects originated by changes in microbiota composition, especially on target bacterial species associated with gut inflammation.

Enzymatic mechanisms for the degradation of phloroglucinol into short-chain fatty acids (SCFAs) have been described in diverse gut bacteria.^40^ Notably, we found that the protective effect of phloroglucinol was not associated with changes in the levels of microbiota-derived SCFAs that have been described as responsible for the protective effect of polyphenolic compounds on DSS-induced pathology in mice.^41^ Indeed, in the DSS-treated groups fecal SCFAs levels were not significantly different between phloroglucinol-treated and control animals (Figure 3(g)). Overall, these data show that phloroglucinol induces minimal changes in microbiota composition and its metabolic response and point to an extraintestinal effect of the metabolite as the main origin of its immunomodulatory properties.

### Phloroglucinol induces trained immunity in vitro and in vivo

**Figure 4.**
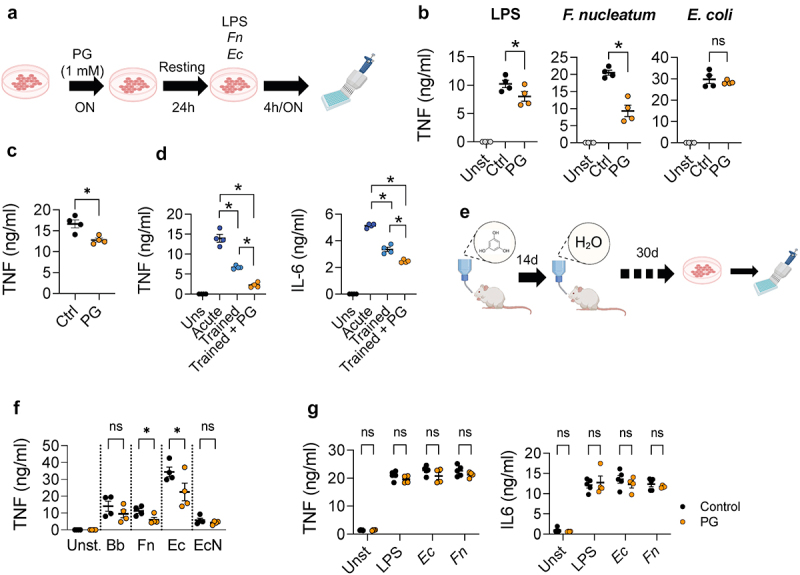
Phloroglucinol induces innate immune training. (a) Experimental design: BMMs were initially stimulated overnight (ON) with phloroglucinol and were left to rest for 24 hours. The macrophages were then stimulated with LPS, *F. nucleatum* and *E. coli* (b) TNF production by BMMs, pre-exposed (orange) or not (black) to phloroglucinol (PG) and stimulated with LPS, *F. nucleatum* and *E. coli*. The grey dots represent unstimulated cells. (c) TNF production by BMMs, pre-exposed (orange) or not (black) to phloroglucinol (PG) and stimulated with *B. burgdorferi*. (d) TNF and IL-6 production by BMMs acutely stimulated (purple) or re-stimulated with *B. burgdorferi* in the presence (orange) or absence of phloroglucinol. (e) Experimental design: mice were administered phloroglucinol for 14 days, followed by 30 days of no supplemented water. BMMs were then generated and stimulated. (f) TNF produced by BMMs from phloroglucinol-treated (orange) and control (black) mice in response to stimulation with *B. burgdorferi* (Bb), *F. nucleatum* (Fn), a clinical *E. coli* isolate (Ec) and *E. coli Nissle 1917* (En). (g) TNF and IL-6 release by BMMs obtained from germ-free mice pre-treated with phloroglucinol (orange) and controls (black), and stimulated with LPS, *F. nucleatum* and *E. coli*.

We hypothesized that the protective effect induced by phloroglucinol is mediated by its interaction with innate immune cells as described for other microbiota-derived metabolites.^2^ To study the capacity of phloroglucinol to imprint attenuated response patterns in macrophages, BMMs were generated and treated overnight with phloroglucinol. After a resting period of 24 hours in the absence of the phenolic compound, macrophages were exposed to inflammatory insults as before (Figure 4(a)). Macrophages previously exposed to phloroglucinol showed attenuated TNF production in response to LPS and *F. nucleatum* (Figure 4(b)).

We also tested the ability of phloroglucinol to affect secondary responses to *B. burgdorferi*. Pre-exposing BMMs to phloroglucinol resulted in a modest, but significant, reduction in TNF production in response to *B. burgdorferi* (Figure 4(c)). Phloroglucinol also diminished TNF and IL-6 production when co-administered with *B. burgdorferi* to trained macrophages^16^ previously exposed to the spirochete (Figure 4(d)). These results showed that phloroglucinol attenuates the inflammatory response of trained macrophages and can induce a trained-like phenotype in these cells. Pre-exposure to phloroglucinol produced variable cell TNF responses that were dependent of the nature of the secondary stimulus, confirming that different PAMPs and particularly, whole bacteria engage different PRRs, greatly influencing the primary and secondary responses of macrophages.^16,42,43^

To determine whether phloroglucinol induces trained immunity *in vivo*, we treated germ-free and conventionally housed mice with the phenolic compound orally for two weeks. The mice were sacrificed after one month of discontinuing the treatment (Figure 4(e)). BMMs were then generated and stimulated with pro-inflammatory insults, as before. BMMs from conventional treated mice showed a general attenuated response to the pro-inflammatory bacteria tested except in the case of *B. burgdorferi* (Figure 4(f)), suggesting that phloroglucinol induces central trained immunity leading to less responsive macrophages. The probiotic strain of *E. coli Nissle 1917* was used as a control because of its generally considered non-inflammatory status. BMMs challenged with this strain did not show changes linked to phloroglucinol treatment. These data show the induction of trained immunity by phloroglucinol that conduces to less responsive macrophages, and results in the prevention of inflammatory responses. Interestingly, BMMs generated from germ-free mice previously treated with phloroglucinol did not show reduced levels of TNF or IL-6 in response to LPS and pro-inflammatory bacteria, in contrast to conventional mice (Figure 4(g)). These results support the requirement of microbiota for the induction of trained immunity in response to phloroglucinol.

### Phloroglucinol induces central trained immunity

**Figure 5.**
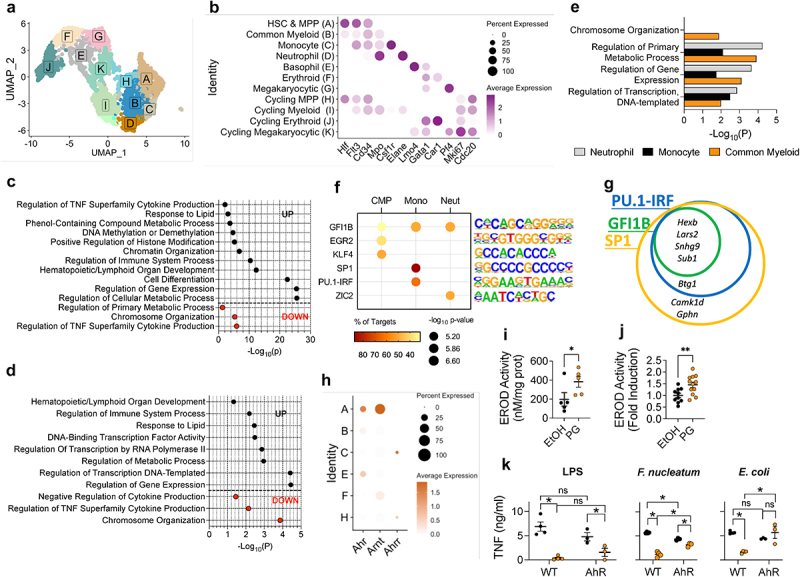
Phloroglucinol induces central trained immunity. (a) UMAP embedding of 3,217 sorted Lin^–^ cKit^+^ hematopoietic stem and progenitor cells from control and phloroglucinol-treated animals. (b) Dot plot showing average expression of selected HSPC markers and cell cycle genes. The dot size corresponds to the fraction of cells expressing each gene, and the dot color intensity represents mean expression values. Pathway analysis (GO-BP) of differentially expressed genes (DEGs) within all cells in the dataset (c), cluster a (d), and clusters B-D (e). (f) Transcription factor motifs associated with upregulated genes in restricted myeloid progenitors. (g) Venn diagram showing genes co- or differentially regulated by the transcription factors GFI1B, SP1, and PU.1-IRF. HSC, hematopoietic stem cell; MPP, multipotent progenitor. (h) Dot plot showing average expression of *Ahr*, *Arnt*, and *Ahrr* in selected clusters. The dot size corresponds to the fraction of cells expressing each gene, and the dot color intensity represents the mean expression value. (i) CYP1A1 enzyme activity (EROD, ethoxyresorufin-O-deethylase) in BMMs stimulated with 1 mM phloroglucinol or vehicle (ethanol) for 24 h. (j) CYP1A1 enzyme activity in colonic tissues of mice given phloroglucinol or vehicle. Data are from two independent experiments and shown as fold induction compared to their respective controls. (k) TNF production by BMMs generated from wild type and AhR-deficient mice. The mice were treated with phloroglucinol as described in Figure 4(f). ns, not significant, *, *p* < 0.05.

To investigate the long-term effects of phloroglucinol treatment at the site of immune cell generation, we performed transcriptomic analysis at single cell resolution on bone marrow hematopoietic stem and progenitor cells (HSPCs). One month after treatment, we sorted and pooled lineage-negative, c-Kit-positive cells from phloroglucinol-treated and control mice to perform single-cell RNA sequencing (scRNAseq). Each group underwent preprocessing, which included filtration, doublet removal, cell cycle scoring, and normalization; following integration, 2,138 and 1,079 cells were captured from phloroglucinol-treated and control mice, respectively. Clustering revealed 11 cell populations, and cluster identity was manually assigned using known hematopoietic markers^31^ and cell cycle genes^44^ (Figure 5(a,b)).

Analysis of differentially expressed genes (DEGs, FDR < 0.05) revealed diverse perturbations. The two most upregulated genes, both by cluster and by all cells in each group, were *Camk1d* and *Hexb* (Figure S4). Pathway analysis of DEGs between all cells in each group revealed alterations in metabolic and epigenetic pathways, implicating trained immunity-related processes, as well as enhancement of hematopoietic- and immune-related processes (Figure 5(c)). Strikingly, upregulation of the pathway “Phenol-Containing Compound Metabolic Process” remained for one month after treatment. When examined by cluster, stem and multipotent progenitors experienced upregulation in gene expression and metabolic pathways, whereas chromosomal and cytokine production pathways were downregulated (Figure 5(d)). Restricted myeloid progenitors retained upregulation in pathways related to metabolism and gene expression, particularly “Regulation of Transcription, DNA-Templated” (Figure 5(e)). Interestingly, the “Chromosome Organization” pathway was significant in the common progenitor but not in the committed precursors, suggesting that changes in epigenetic processes do not play a role following lineage commitment.

Lingering trained immunity effects through generations rely on epigenetic modifications and changes in transcription factor activity.^45^ To explore differentially regulated transcription factor activity among myeloid progenitors, phloroglucinol-induced upregulated DEGs from the common myeloid, monocyte, and neutrophil progenitors (clusters B, C, and D) underwent transcription factor enrichment analysis with HOMER. Several transcription factors were putatively responsible for the transcriptional shifts in phloroglucinol-treated animals, with GFI1B being commonly enriched between these myeloid clusters (Figure 5(f)). Within the monocyte progenitors, comparison of the DEGs regulated by GFI1B, SP1, and PU.1-IRF revealed four commonly regulated genes: *Hexb*, *Lars2*, *Snhg9*, and *Sub1* (Figure 5(g), Figure S4).

The aryl hydrocarbon receptor (AhR) has been associated with the response of immune and other cells to some diet-derived metabolites.^46–48^ Therefore, we analyzed the expression of *Ahr* and the gene coding for its coactivator, HIF1B (*Arnt*) within HSPCs. Coexpression of both genes was only detected in the most quiescent population of hematopoietic stem cells (HSCs) and Multipotent Progenitors (MPPs) (Figure 5(h)). Interestingly, the expression of the gene coding for the AHR repressor, AHRR (*Ahrr*) was not detected in this population (Figure 5(h)), overall pointing to the most undifferentiated cells as the target of phloroglucinol. The exposure of BMMs to phloroglucinol resulted in increased EROD activity (Figure 5(i)), while the oral treatment with the compound also increased EROD activity in the colonic tissue (Figure 5(j)), confirming AhR as a bona fide phloroglucinol receptor. Notably, the absence of AhR largely abolished the effect of phloroglucinol on proinflammatory bacteria-induced TNF production, most predominantly, *E. coli*, while its anti-inflammatory effect was still noticeable in response to LPS and *F. nucleatum*, albeit significantly reduced in the latter case (Figure 5(k)). These data showed that AhR is, at least in part, a mediator of the activity of phloroglucinol.

Taken together, these results demonstrate the lingering transcriptional shift effects of phloroglucinol treatment in bone marrow HSPC populations.

### Phloroglucinol induces long-term protection in intra- and extraintestinal murine models of inflammatory disorders

**Figure 6.**
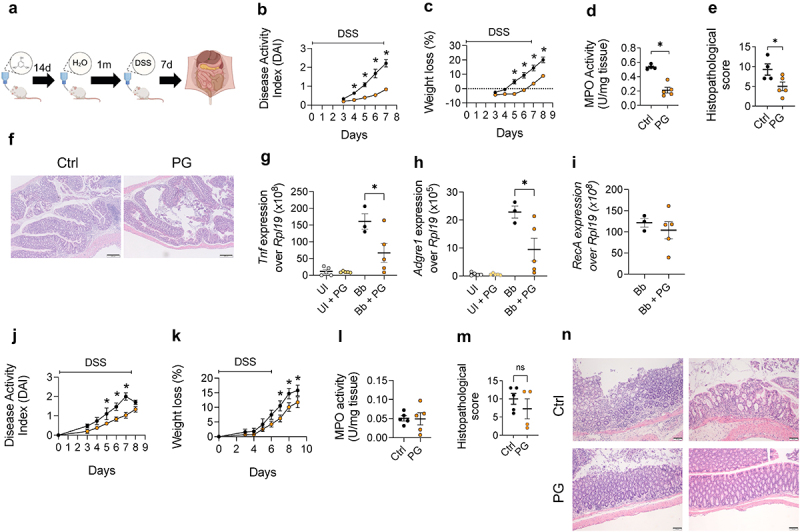
Phloroglucinol induces long-term protection in intra- and extra-intestinal murine models of inflammatory disorders. (a) Experimental design: mice were treated for 14 days with phloroglucinol in the drinking water, followed by 30 days of resting. The mice were then induced IBD with a 3% solution of DSS for 6 days, followed by 2 days of resting. DAI (b) and weight loss (c) over the experimental period in DSS-induced mice, phloroglucinol-treated mice (orange) and controls (black). Data are expressed as means ± sem (*n* = 4–5); *p < 0.05 versus DSS control group. (d) MPO activity in mice treated with phloroglucinol (orange) and controls (black). Histopathological scores (e) and representative micrographs (f) of colonic tissue of mice treated with phloroglucinol (orange) and controls (black). *Tnf* (g), *Adgre1* (h) gene expression and *B. burgdorferi recA* (i) DNA levels relative to murine *Rpl19* in the heart of phloroglucinol-treated, infected mice (orange), control-treated infected animals (black) and uninfected mice treated (yellow) or not (grey) with phloroglucinol as determined by cDNA real-time PCR relative to *Rpl19*. DAI (j) and weight loss (k) of mice treated with phloroglucinol for 14 days, followed by a resting period of six months. The mice were then induced IBD with 3% DSS in sterile water for 6 days, followed by 2 days of resting. The orange dots represent the DSS-induced, phloroglucinol-treated mice. The black dots represent DSS-induced controls. The data are expressed as means ± sem (*n* = 5); *p < 0.05 versus DSS control group. (l) MPO activity in mice treated with phloroglucinol (orange) and controls (black), and rested for 6 months. Histopathological scores (m) and representative micrographs (n) of colonic tissue in mice treated with phloroglucinol (orange) and controls (black), and rested for 6 months.

To determine whether the long-term effect of phloroglucinol on BMMs translated into lasting *in vivo* anti-inflammatory protection, we investigated DSS- and *B. burgdorferi*-induced inflammation in mice that had been previously treated with the phenolic compound for two weeks, followed by a one month resting period (Figure 6(a)). Compared to control animals, phloroglucinol-treated mice showed a potent reduction in several parameters after DSS treatment, including DAI (Figure 6(b)), weight loss (Figure 6(c)), MPO levels (Figure 6(d)) and global histopathological scores (Figure 6(e,f)), exhibiting long-lasting anti-inflammatory effects during gut inflammation.

A similar long-term protection effect was observed when mice were infected with *B. burgdorferi* one month after phloroglucinol treatment. We observed a significant reduction in *Tnf* gene expression and macrophage infiltration in the heart of mice previously treated with phloroglucinol compared to control animals (Figure 6(g,h)). The bacterial burdens in the cardiac tissue were not affected by the treatment (Figure 6(i)), confirming a specific effect of phloroglucinol on the pro-inflammatory capacity of macrophages without affecting their phagocytic activity.

To assess whether the effect of phloroglucinol could extend beyond one month after treatment, we treated a group of mice with the phenolic compound, as before, and let them rest for six months. Remarkably, the anti-inflammatory effect of phloroglucinol was still evident when the mice were induced colon inflammation with DSS after the 6-month resting period. The treatment resulted in the reduction in DAI (Figure 6(j)) and weight loss (Figure 6(k)). However, no difference in MPO levels were observed at sacrifice (Figure 6(l)). At the histological level, the effect of the pretreatment with phloroglucinol was still partially evident in some of the mice, although the analysis of the histopathological scores did not show significant differences between groups (Figure 6(m,n)). These results showed that phloroglucinol administration can provide long-lasting protective effects in a murine model of gut inflammation, particularly in weight loss, at least for a resting period equivalent to a quarter of the mouse life.

## Discussion

Several dietary polyphenols are poorly absorbed and reach the colon, where the intestinal microbiota metabolizes them, rendering bioactive metabolites. We explored the immunomodulatory effects of phloroglucinol because it is a common microbial metabolite obtained from many dietary polyphenols,^6^ and it is being used clinically for IBS treatment.^12,13^ Phloroglucinol pretreatment alleviated DSS-induced colitis, similarly to what has been observed with other phenolic compounds.^41,49,50^ This capacity is often associated with an increased abundance of *A. muciniphila*.^41^ Unlike previous findings with other phenolic compounds,^41,49,50^ however, we observed neither drastic shifts in microbiota composition nor significant changes in the abundance of *A. muciniphila*, despite the phloroglucinol-induced growth enhancement of this bacterium *in vitro*. Despite the absence of major changes in its composition after phloroglucinol administration, the microbiota was necessary to trigger phloroglucinol induction of long-term protection. In contrast to their conventional counterparts, BMMs obtained from germ-free animals did not present attenuated responses to pro-inflammatory pathogens, which suggests that punctual microbiota changes might be relevant for the induction of central training. Alternatively, a general need for microbiota in the induction of long-term central training cannot be discarded.

Metabolically, the increase of gut microbial-produced SCFAs has been postulated to explain the protection against DSS-induced damage of phenolic compounds.^41^ A complete degradative pathway for phloroglucinol transformation yielding SCFAs that involves a variety of gut microbiota taxa has been recently reported.^40^ However, we did not observe significant changes in SCFA levels associated with the treatment. In contrast, we observed the capacity of phloroglucinol to reach the blood and urine, which led us to explore its effect on the pathogenesis of the non-intestinal pathogen *B. burgdorferi*. Treatment during acute infection dramatically reduced macrophage infiltration and inflammation, while pretreatment one month before induced similar effects. This prophylactic effect was recapitulated in the DSS model that remarkably partially extended for up to six months. Studies addressing the stability and malleability of the training effects produced by phloroglucinol need to be performed to understand its properties in environments less stable than animal facilities. These will also unveil whether repeated exposure to the metabolite cements its protective effect or rather, further modulates its immune modulatory capacity. On the other hand, given the different characteristics of the immune response and microbiota between mice and humans, the effect of the metabolite on human immune modulation might be different. For example, phloroglucinol might be differently transformed by microbes exclusively present in humans and mice as recently suggested for other related phenolic compounds.^51^ Therefore, addressing the effect of phloroglucinol on the human microbiota and the capacity of human gut microorganisms to transform the metabolite will allow a more accurate prediction of its immune modulatory capacity in humans. Overall, the long-term protective effects of phloroglucinol point to its capacity to induce trained immunity and therefore, modulate macrophage activity that drives pathogenesis.

A trained immunity-related phenotype was confirmed by the transcriptomic analysis of HSPCs, which revealed alterations in metabolic and epigenetic pathways, and that were most prevalent in upstream progenitors. Because maintenance of trained immunity during cell division depends on continuous transcription factor activity paired with epigenetic changes,^45^ we identified regulators responsible for the transcriptomic shifts in myeloid HSPCs one month after treatment, such as enhanced GFI1B activity. Despite normal expression in less than 10% of granulomonocytic precursors,^52^ GFI1B serves as a metabolic regulator of hematopoiesis through epigenetic control of fatty acid oxidation genes.^53^ Consistently, we observed upregulation of the “Response to Lipid” pathway in the uncommitted HSPC populations. Furthermore, GFI1B and other identified transcription factors regulate *Snhg9* expression, a long non-coding RNA exploited by gut microbiota to modulate lipid metabolism.^54^ Together, these data describe the lingering transcriptional modulation of HSPCs following phloroglucinol treatment, which is likely responsible for the protective phenotype in myeloid-derived cells.

We propose that phloroglucinol acts through AhR, a receptor for gut-derived phenolic compounds.^33^ Coexpression of *Ahr* and its cofactor *Arnt* (HIF1B) was restricted to upstream progenitors. Most notably, BMMs from *Ahr*-deficient mice did not respond to phloroglucinol pretreatment, while AhR activity was readily detected both in BMMs treated with the metabolite and in the colon tissue of phloroglucinol-treated mice. AhR regulates steady-state hematopoiesis and affects lineage potential,^55^ and AhR-mediated signaling depends on calcium/calmodulin (CaM)-dependent protein kinase (CaMK) pathways.^56^ It is likely that AhR-induced signals are no longer relevant after 1 month of discontinuing the treatment. Indeed, our analysis failed to identify AhR-induced gene expression upregulation. Therefore, the continuous upregulation of *Camk1d*, which seems to be controlled by SP1, may suggest AhR-independent activity of CAMK that is still involved in the long-term effects initiated by phloroglucinol.

These results represent the characterization of a specific, orally administered microbiota-derived metabolite capable of inducing durable central trained immunity *in vivo*. Previous studies have demonstrated that microbiota-derived metabolites can confer protection against diverse inflammatory processes. For example, the intravenous administration of a microbiota-transformed bile acid (deoxycholic acid) protects against intestinal amebiasis through expansion of bone marrow granulocyte-monocyte progenitors^57^ in a vitamin D receptor-dependent manner,^58^ although macrophage training was not explicitly demonstrated. On the other hand, the oral treatment with butyrate resulted in trained colonic macrophages without affecting BM progenitors.^2^ Conversely, after murine BCG vaccination, gut-derived metabolites, such as L-carnitine derivatives produced during mycobacterial-induced dysbiosis, train tissue resident alveolar macrophages.^59^ However, L-carnitine and SCFA coadministration in drinking water trained alveolar macrophages but not circulating monocytes, demonstrating specific peripheral, but not central, training.^59^ Interestingly, the human BCG-induced trained phenotype correlates with specific microbiota taxa and metabolic processes at baseline,^60^ suggesting that unique microbial metabolic pathways contribute to the trained immunity process.

The origin of intestinal phloroglucinol is not fully understood. Although not common, food components, such as garden onion and tea, contain phloroglucinol. However, fecal metabolomics studies have detected a pronounced increase in the metabolite levels after ingestion of phenolic-rich foods, with high individual variability,^10,61^ further supporting the differential contribution of individual microbiota to gut phloroglucinol levels. Moreover, different phloroglucinol-related pathways (synthetic and degradative) have been suggested.^62,63^ Overall, our results demonstrate the modulation of host immunity with a microbiota-derived molecule derived from fruit and vegetable consumption that confers protective effects across disease states. They also represent an evolution in our functional understanding of the host-microbiota interface and their therapeutic potential for diverse inflammatory pathologies.

## Supplementary Material

Suppl_Info.docx

## References

[1] Tomas-Barberan FA, Selma MV, Espin JC. Interactions of gut microbiota with dietary polyphenols and consequences to human health. Curr Opin Clin Nutr Metab Care. 2016;19(6):471–22. doi:10.1097/MCO.0000000000000314. 27490306

[2] Schulthess J, Pandey S, Capitani M, Rue-Albrecht KC, Arnold I, Franchini F, Chomka A, Ilott NE, Johnston DGW, Pires E, et al. The short chain fatty acid butyrate imprints an antimicrobial program in macrophages. Immunity. 2019;50(2):432–45 e7. doi:10.1016/j.immuni.2018.12.018. 30683619 PMC6382411

[3] Oh SF, Praveena T, Song H, Yoo JS, Jung DJ, Erturk-Hasdemir D, Hwang YS, Lee CC, Le Nours J, Kim H, et al. Host immunomodulatory lipids created by symbionts from dietary amino acids. Nature. 2021;600(7888):302–307. doi:10.1038/s41586-021-04083-0. 34759313 PMC8999822

[4] Steed AL, Christophi GP, Kaiko GE, Sun L, Goodwin VM, Jain U, Esaulova E, Artyomov MN, Morales DJ, Holtzman MJ, et al. The microbial metabolite desaminotyrosine protects from influenza through type I interferon. Science. 2017;357(6350):498–502. doi:10.1126/science.aam5336. 28774928 PMC5753406

[5] Stutz MR, Dylla NP, Pearson SD, Lecompte-Osorio P, Nayak R, Khalid M, Adler E, Boissiere J, Lin H, Leiter W, et al. Immunomodulatory fecal metabolites are associated with mortality in COVID-19 patients with respiratory failure. Nat Commun. 2022;13(1):6615. doi:10.1038/s41467-022-34260-2. 36329015 PMC9633022

[6] Selma MV, Espin JC, Tomas-Barberan FA. Interaction between phenolics and gut microbiota: role in human health. J Agric Food Chem. 2009;57(15):6485–6501. doi:10.1021/jf902107d. 19580283

[7] Liang A, Leonard W, Beasley JT, Fang Z, Zhang P, Ranadheera CS. Anthocyanins-gut microbiota-health axis: a review. Crit Rev Food Sci Nutr. 2023;64(21):7563–7588. 10.1080/10408398.2023.2187212. 36927343

[8] Zhang Z, Li S, Cao H, Shen P, Liu J, Fu Y, Cao Y, Zhang N. The protective role of phloretin against dextran sulfate sodium-induced ulcerative colitis in mice. Food Funct. 2019;10(1):422–431. doi:10.1039/C8FO01699B. 30604787

[9] Blasco T, Perez-Burillo S, Balzerani F, Hinojosa-Nogueira D, Lerma-Aguilera A, Pastoriza S, Cendoya X, Rubio Á, Gosalbes MJ, Jiménez-Hernández N, et al. An extended reconstruction of human gut microbiota metabolism of dietary compounds. Nat Commun. 2021;12(1):4728. doi:10.1038/s41467-021-25056-x. 34354065 PMC8342455

[10] Gill CI, McDougall GJ, Glidewell S, Stewart D, Shen Q, Tuohy K, Dobbin A, Boyd A, Brown E, Haldar S, et al. Profiling of phenols in human fecal water after raspberry supplementation. J Agric Food Chem. 2010;58(19):10389–10395. doi:10.1021/jf1017143. 20809621

[11] Marasinghe CK, Jung WK, Je JY. Phloroglucinol possesses anti-inflammatory activities by regulating AMPK/Nrf2/HO-1 signaling pathway in lps-stimulated RAW264.7 murine macrophages. Immunopharmacol Immunotoxicol. 2023;45(5):571–580. doi:10.1080/08923973.2023.2196602. 36988555

[12] Chassany O, Bonaz B, Bruley DES Varannes S, Bueno L, Cargill G, Coffin B, Ducrotté P, Grangé V. Acute exacerbation of pain in irritable bowel syndrome: efficacy of phloroglucinol/trimethylphloroglucinol – a randomized, double-blind, placebo-controlled study. Aliment Pharmacol Ther. 2007;25(9):1115–1123. doi:10.1111/j.1365-2036.2007.03296.x. 17439513 PMC2683251

[13] Shin SY, Cha BK, Kim WS, Park JY, Kim JW, Choi CH. The effect of phloroglucinol in patients with diarrhea-predominant irritable bowel syndrome: a randomized, double-blind, placebo-controlled trial. J Neurogastroenterol Motil. 2020;26(1):117–127. doi:10.5056/jnm19160. 31917916 PMC6955199

[14] Smith K, McCoy KD, Macpherson AJ. Use of axenic animals in studying the adaptation of mammals to their commensal intestinal microbiota. Semin Immunol. 2007;19(2):59–69. doi:10.1016/j.smim.2006.10.002. 17118672

[15] Schmidt JV, Su GH, Reddy JK, Simon MC, Bradfield CA. Characterization of a murine Ahr null allele: involvement of the Ah receptor in hepatic growth and development. Proc Natl Acad Sci USA. 1996;93(13):6731–6736. doi:10.1073/pnas.93.13.6731. 8692887 PMC39095

[16] Barriales D, Martin-Ruiz I, Carreras-Gonzalez A, Montesinos-Robledo M, Azkargorta M, Iloro I, Escobés I, Martín-Mateos T, Atondo E, Palacios A, et al. Borrelia burgdorferi infection induces long-term memory-like responses in macrophages with tissue-wide consequences in the heart. PLOS Biol. 2021;19(1):e3001062. doi:10.1371/journal.pbio.3001062. 33395408 PMC7808612

[17] Li XQ, Wang RT, Wang QH, Tang XL, Lu CT, Gong HG, Wen A-D. Determination of phloroglucinol by HPLC-MS/MS and its application to a bioequivalence study in healthy volunteers. Eur Rev Med Pharmacol Sci. 2017;21(8):1990–1998. 28485775

[18] Camuesco D, Comalada M, Rodriguez-Cabezas ME, Nieto A, Lorente MD, Concha A, Zarzuelo A, Gálvez J. The intestinal anti-inflammatory effect of quercitrin is associated with an inhibition in iNOS expression. Br J Pharmacol. 2004;143(7):908–918. doi:10.1038/sj.bjp.0705941. 15533892 PMC1575937

[19] Pascual-Itoiz MA, Pena-Cearra A, Martin-Ruiz I, Lavin JL, Simo C, Rodriguez H, Atondo E, Flores JM, Carreras-González A, Tomás-Cortázar J, et al. The mitochondrial negative regulator MCJ modulates the interplay between microbiota and the host during ulcerative colitis. Sci Rep. 2020;10(1):572. doi:10.1038/s41598-019-57348-0. 31953445 PMC6969106

[20] Schloss PD. Reintroducing mothur: 10 Years Later. Appl Environ Microbiol. 2020;86(2). doi:10.1128/AEM.02343-19. PMC6952234 31704678

[21] Schloss PD, Westcott SL, Ryabin T, Hall JR, Hartmann M, Hollister EB, Lesniewski RA, Oakley BB, Parks DH, Robinson CJ, et al. Introducing mothur: open-source, platform-independent, community-supported software for describing and comparing microbial communities. Appl Environ Microbiol. 2009;75(23):7537–7541. doi:10.1128/AEM.01541-09. 19801464 PMC2786419

[22] Kozich JJ, Westcott SL, Baxter NT, Highlander SK, Schloss PD. Development of a dual-index sequencing strategy and curation pipeline for analyzing amplicon sequence data on the MiSeq illumina sequencing platform. Appl Environ Microbiol. 2013;79(17):5112–5120. doi:10.1128/AEM.01043-13. 23793624 PMC3753973

[23] Quast C, Pruesse E, Yilmaz P, Gerken J, Schweer T, Yarza P, Peplies J, Glöckner FO. The SILVA ribosomal RNA gene database project: improved data processing and web-based tools. Nucleic Acids Res. 2013;41(Database issue):D590–6. doi:10.1093/nar/gks1219. 23193283 PMC3531112

[24] McMurdie PJ, Holmes S. Phyloseq: an R package for reproducible interactive analysis and graphics of microbiome census data. PLOS ONE. 2013;8(4):e61217. doi:10.1371/journal.pone.0061217. 23630581 PMC3632530

[25] Love MI, Huber W, Anders S. Moderated estimation of fold change and dispersion for RNA-seq data with DESeq2. Genome Biol. 2014;15(12):550. doi:10.1186/s13059-014-0550-8. 25516281 PMC4302049

[26] Han J, Lin K, Sequeira C, Borchers CH. An isotope-labeled chemical derivatization method for the quantitation of short-chain fatty acids in human feces by liquid chromatography–tandem mass spectrometry. Anal Chim Acta. 2015;854:86–94. doi:10.1016/j.aca.2014.11.015. 25479871

[27] Zheng GX, Terry JM, Belgrader P, Ryvkin P, Bent ZW, Wilson R, Ziraldo SB, Wheeler TD, McDermott GP, Zhu J, et al. Massively parallel digital transcriptional profiling of single cells. Nat Commun. 2017;8(1):14049. doi:10.1038/ncomms14049. 28091601 PMC5241818

[28] Hao Y, Hao S, Andersen-Nissen E, Mauck WM 3rd, Zheng S, Butler A, Lee MJ, Wilk AJ, Darby C, Zager M, et al. Integrated analysis of multimodal single-cell data. Cell. 2021;184(13):3573–87 e29. doi:10.1016/j.cell.2021.04.048. 34062119 PMC8238499

[29] McGinnis CS, Murrow LM, Gartner ZJ. DoubletFinder: doublet detection in single-cell RNA sequencing data using artificial nearest neighbors. Cell Syst. 2019;8(4):329–37 e4. doi:10.1016/j.cels.2019.03.003. 30954475 PMC6853612

[30] Tirosh I, Venteicher AS, Hebert C, Escalante LE, Patel AP, Yizhak K, Fisher JM, Rodman C, Mount C, Filbin MG, et al. Single-cell RNA-seq supports a developmental hierarchy in human oligodendroglioma. Nature. 2016;539(7628):309–313. doi:10.1038/nature20123. 27806376 PMC5465819

[31] Konturek-Ciesla A, Dhapola P, Zhang Q, Sawen P, Wan H, Karlsson G, Bryder D. Temporal multimodal single-cell profiling of native hematopoiesis illuminates altered differentiation trajectories with age. Cell Rep. 2023;42(4):112304. doi:10.1016/j.celrep.2023.112304. 36961818

[32] Heinz S, Benner C, Spann N, Bertolino E, Lin YC, Laslo P, Cheng JX, Murre C, Singh H, Glass CK. Simple combinations of lineage-determining transcription factors prime cis-regulatory elements required for macrophage and B cell identities. Mol Cell. 2010;38(4):576–589. doi:10.1016/j.molcel.2010.05.004. 20513432 PMC2898526

[33] Rothhammer V, Quintana FJ. The aryl hydrocarbon receptor: an environmental sensor integrating immune responses in health and disease. Nat Rev Immunol. 2019;19(3):184–197. doi:10.1038/s41577-019-0125-8. 30718831

[34] Schiering C, Wincent E, Metidji A, Iseppon A, Li Y, Potocnik AJ, Omenetti S, Henderson CJ, Wolf CR, Nebert DW, et al. Feedback control of AHR signalling regulates intestinal immunity. Nature. 2017;542(7640):242–245. doi:10.1038/nature21080. 28146477 PMC5302159

[35] Hume DA, Ross IL, Himes SR, Sasmono RT, Wells CA, Ravasi T. The mononuclear phagocyte system revisited. J Leukoc Biol. 2002;72(4):621–627. doi:10.1189/jlb.72.4.621. 12377929

[36] Scott SA, Fu J, Chang PV. Microbial tryptophan metabolites regulate gut barrier function via the aryl hydrocarbon receptor. Proc Natl Acad Sci USA. 2020;117(32):19376–19387. doi:10.1073/pnas.2000047117. 32719140 PMC7431026

[37] Brinkman BM, Becker A, Ayiseh RB, Hildebrand F, Raes J, Huys G, Vandenabeele P. Gut microbiota affects sensitivity to acute dss-induced colitis independently of host genotype. Inflamm Bowel Dis. 2013;19(12):2560–2567. doi:10.1097/MIB.0b013e3182a8759a. 24105395

[38] Guo X, Huang C, Xu J, Xu H, Liu L, Zhao H, Wang J, Huang W, Peng W, Chen Y, et al. Gut microbiota is a potential biomarker in inflammatory bowel disease. Front Nutr. 2021;8:818902. doi:10.3389/fnut.2021.818902. 35127797 PMC8814525

[39] Iljazovic A, Roy U, Galvez EJC, Lesker TR, Zhao B, Gronow A, Amend L, Will SE, Hofmann JD, Pils MC, et al. Perturbation of the gut microbiome by Prevotella spp. enhances host susceptibility to mucosal inflammation. Mucosal Immunol. 2021;14(1):113–124. doi:10.1038/s41385-020-0296-4. 32433514 PMC7790746

[40] Zhou Y, Wei Y, Jiang L, Jiao X, Zhang Y, Harwood CS. Anaerobic phloroglucinol degradation by Clostridium scatologenes. mBio. 2023;14(4):e0109923. doi:10.1128/mbio.01099-23. 37341492 PMC10470551

[41] Wu Z, Huang S, Li T, Li N, Han D, Zhang B, Xu ZZ, Zhang S, Pang J, Wang S, et al. Gut microbiota from green tea polyphenol-dosed mice improves intestinal epithelial homeostasis and ameliorates experimental colitis. Microbiome. 2021;9(1):184. doi:10.1186/s40168-021-01115-9. 34493333 PMC8424887

[42] Pellon A, Barriales D, Pena-Cearra A, Castelo-Careaga J, Palacios A, Lopez N, Atondo E, Pascual-Itoiz MA, Martín-Ruiz I, Sampedro L, et al. The commensal bacterium Lactiplantibacillus plantarum imprints innate memory-like responses in mononuclear phagocytes. Gut Microbes. 2021;13(1):1939598. doi:10.1080/19490976.2021.1939598. 34224309 PMC8259724

[43] Pena-Cearra A, Palacios A, Pellon A, Castelo J, Pasco ST, Seoane I, Barriales D, Martin JE, Pascual-Itoiz MÁ, Gonzalez-Lopez M, et al. Akkermansia muciniphila-induced trained immune phenotype increases bacterial intracellular survival and attenuates inflammation. Commun Biol. 2024;7(1):192. doi:10.1038/s42003-024-05867-6. 38365881 PMC10873422

[44] Tirosh I, Izar B, Prakadan SM, Wadsworth MH 2nd, Treacy D, Trombetta JJ, Rotem A, Rodman C, Lian C, Murphy G, et al. Dissecting the multicellular ecosystem of metastatic melanoma by single-cell RNA-seq. Science. 2016;352(6282):189–196. doi:10.1126/science.aad0501. 27124452 PMC4944528

[45] Sun S, Aguirre-Gamboa R, Barreiro LB. Transmission of stimulus-induced epigenetic changes through cell division is coupled to continuous transcription factor activity. Front Immunol. 2023;14:1129577. doi:10.3389/fimmu.2023.1129577. 36999024 PMC10043173

[46] Hubbard TD, Murray IA, Perdew GH. Indole and tryptophan metabolism: endogenous and dietary routes to Ah receptor activation. Drug Metab Dispos. 2015;43(10):1522–1535. doi:10.1124/dmd.115.064246. 26041783 PMC4576673

[47] Amakura Y, Tsutsumi T, Sasaki K, Nakamura M, Yoshida T, Maitani T. Influence of food polyphenols on aryl hydrocarbon receptor-signaling pathway estimated by in vitro bioassay. Phytochemistry. 2008;69(18):3117–3130. doi:10.1016/j.phytochem.2007.07.022. 17869316

[48] Pinto CJG, Avila-Galvez MA, Lian Y, Moura-Alves P, Nunes Dos Santos C. Targeting the aryl hydrocarbon receptor by gut phenolic metabolites: a strategy towards gut inflammation. Redox Biol. 2023;61:102622. doi:10.1016/j.redox.2023.102622. 36812782 PMC9958510

[49] Zhang Z, Wu X, Cao S, Wang L, Wang D, Yang H, Feng Y, Wang S, Li L. Caffeic acid ameliorates colitis in association with increased akkermansia population in the gut microbiota of mice. Oncotarget. 2016;7(22):31790–31799. doi:10.18632/oncotarget.9306. 27177331 PMC5077976

[50] Wei Y, Gao J, Kou Y, Liu M, Meng L, Zheng X, Xu S, Liang M, Sun H, Liu Z, et al. The intestinal microbial metabolite desaminotyrosine is an anti-inflammatory molecule that modulates local and systemic immune homeostasis. Faseb J. 2020;34(12):16117–16128. doi:10.1096/fj.201902900RR. 33047367

[51] Culp EJ, Nelson NT, Verdegaal AA, Goodman AL. Microbial transformation of dietary xenobiotics shapes gut microbiome composition. Cell. 2024;187(22):6327–6345.e20. doi:10.1016/j.cell.2024.08.038. 39321800 PMC11531382

[52] Vassen L, Okayama T, Moroy T. Gfi1b: green fluorescent protein knock-in mice reveal a dynamic expression pattern of Gfi1b during hematopoiesis that is largely complementary to Gfi1. Blood. 2007;109(6):2356–2364. doi:10.1182/blood-2006-06-030031. 17095621

[53] Liu L, Patnana PK, Xie X, Frank D, Nimmagadda SC, Su M, Zhang D, Koenig T, Rosenbauer F, Liebmann M, et al. GFI1B acts as a metabolic regulator in hematopoiesis and acute myeloid leukemia. Leukemia. 2022;36(9):2196–2207. doi:10.1038/s41375-022-01635-9. 35804097 PMC9417998

[54] Wang Y, Wang M, Chen J, Li Y, Kuang Z, Dende C, Raj P, Quinn G, Hu Z, Srinivasan T, et al. The gut microbiota reprograms intestinal lipid metabolism through long noncoding RNA Snhg9. Science. 2023;381(6660):851–857. doi:10.1126/science.ade0522. 37616368 PMC10688608

[55] Vaughan KL, Franchini AM, Kern HG, Lawrence BP. The aryl hydrocarbon receptor modulates murine hematopoietic stem cell homeostasis and influences lineage-biased stem and progenitor cells. STEM Cells Dev. 2021;30(19):970–980. doi:10.1089/scd.2021.0096. 34428990 PMC8851211

[56] Monteiro P, Gilot D, Le Ferrec E, Rauch C, Lagadic-Gossmann D, Fardel O. Dioxin-mediated up-regulation of aryl hydrocarbon receptor target genes is dependent on the calcium/calmodulin/CaMKIα pathway. Mol Pharmacol. 2008;73(3):769–777. doi:10.1124/mol.107.043125. 18089838

[57] Burgess SL, Leslie JL, Uddin J, Oakland DN, Gilchrist C, Moreau GB, Watanabe K, Saleh M, Simpson M, Thompson BA, et al. Gut microbiome communication with bone marrow regulates susceptibility to amebiasis. J Clin Invest. 2020;130(8):4019–4024. doi:10.1172/JCI133605. 32369444 PMC7410058

[58] Thompson B, Lu S, Revilla J, Uddin MJ, Oakland DN, Brovero S, Keles S, Bresnick EH, Petri WA, Burgess SL. Secondary bile acids function through the vitamin D receptor in myeloid progenitors to promote myelopoiesis. Blood Adv. 2023;7(17):4970–4982. doi:10.1182/bloodadvances.2022009618. 37276450 PMC10463201

[59] Jeyanathan M, Vaseghi-Shanjani M, Afkhami S, Grondin JA, Kang A, D’Agostino MR, Yao Y, Jain S, Zganiacz A, Kroezen Z, et al. Parenteral BCG vaccine induces lung-resident memory macrophages and trained immunity via the gut–lung axis. Nat Immunol. 2022;23(12):1687–1702. doi:10.1038/s41590-022-01354-4. 36456739 PMC9747617

[60] Strazar M, Mourits VP, Koeken V, de Bree LCJ, Moorlag S, Joosten LAB, van Crevel R, Vlamakis H, Netea MG, Xavier RJ. The influence of the gut microbiome on bcg-induced trained immunity. Genome Biol. 2021;22(1):275. doi:10.1186/s13059-021-02482-0. 34551799 PMC8456614

[61] Gao X, Pujos-Guillot E, Martin JF, Galan P, Juste C, Jia W, Sebedio J-L. Metabolite analysis of human fecal water by gas chromatography/mass spectrometry with ethyl chloroformate derivatization. Anal Biochem. 2009;393(2):163–175. doi:10.1016/j.ab.2009.06.036. 19573517

[62] Brune A, Schink B. Phloroglucinol pathway in the strictly anaerobic Pelobacter acidigallici: fermentation of trihydroxybenzenes to acetate via triacetic acid. Arch Microbiol. 1992;157(5):417–424. doi:10.1007/BF00249098.

[63] Winter J, Moore LH, Dowell VR Jr., Bokkenheuser VD. C-ring cleavage of flavonoids by human intestinal bacteria. Appl Environ Microbiol. 1989;55(5):1203–1208. doi:10.1128/aem.55.5.1203-1208.1989. 2757380 PMC184277

