# Supplementary material for: The microbiota metabolite, phloroglucinol, confers long-term protection against inflammation
Source: Gut Microbes. 2024 Dec 15;16(1):2438829. doi: 10.1080/19490976.2024.2438829 (PMC11651279; doi:10.1080/19490976.2024.2438829)
Supplement: Suppl_Info.docx [file KGMI_A_2438829_SM2601.docx]

Supplementary Information

# The microbiota metabolite, phloroglucinol, confers long-term protection against inflammation

Janire Castelo^1†^, Sarai Araujo-Aris^1†^, Diego Barriales^1†‡^, Samuel Tanner Pasco^1†^, Iratxe Seoane^1,2^, Ainize Peña-Cearra^1,2^, Ainhoa Palacios^1‡^, Carolina Simó^3^, Virginia Garcia-Cañas^3^, Muthita Khamwong^4^, Itziar Martín-Ruiz^1^, Monika Gonzalez-Lopez^1^, Laura Barcena^1^, José Ezequiel Martín Rodríguez^1^, José Luís Lavín^5^, Naiara Gutiez^1^, Raquel Marcos^6,7^, Estibaliz Atondo^1^, Arantza Cobela^1§^, Laura Plaza-Vinuesa^8^, Adrián Plata^1^, Eneko Santos-Fernandez^1^, Alberto Fernandez-Tejada^1,9^, Mari Carmen Villarán^10^, José Miguel Mancheño^11^, Juana Maria Flores^12^, Ana María Aransay^1,13^, Aize Pellón^1^, Blanca de las Rivas^8^, Rosario Muñoz^8^, Abelardo Margolles^6,7^, Patricia Ruas-Madiedo^6,7^, Maria Victoria Selma^14^, Mercedes Gomez de Agüero^4^, Leticia Abecia^1,2^, Juan Anguita^1,9*^ and Héctor Rodríguez^1*^

1CIC bioGUNE, Basque Research and Technology Alliance (BRTA); Derio, Spain. 2Department of Immunology, Microbiology and Parasitology, Faculty of Medicine and Nursing, University of the Basque Country (UPV/EHU); Bilbao, Spain.

3Molecular Nutrition and Metabolism, Institute of Food Science Research (CIAL), Spanish National Research Council (CSIC); Madrid, Spain.

4Würzburg Institute of Systems Immunology, Max-Planck Research Group at the Julius- Maximilians Universität; Würzburg, Germany.

5Applied Mathematics Department - Bioinformatics Unit, NEIKER-BRTA; Derio, Spain.

6Departamento de Microbiología y Bioquímica, Instituto de Productos Lácteos de Asturias, CSIC; Villaviciosa, Spain.

7Functionality and Ecology of Beneficial Microbes (MicroHealth) Group, Instituto de Investigación Sanitaria del Principado de Asturias; Oviedo, Spain.

8Instituto de Ciencia y Tecnología de Alimentos y Nutrición (ICTAN), CSIC; Madrid, Spain.

9Ikerbasque, Basque Foundation for Science; Bilbao, Spain.

10Tecnalia-BRTA; Miñano, Spain.

11Department of Crystallography and Structural Biology, Institute of Physical Chemistry Blas Cabrera (IQF), CSIC; Madrid, Spain.

12Department of Animal Medicine and Surgery, Veterinary Faculty, Universidad Complutense de Madrid; Madrid, Spain.

13CIBERehd, Instituto de Salud Carlos III (ISCIII); Madrid, Spain.

14Laboratory of Food & Health, Research Group on Quality, Safety and Bioactivity of Plant Foods, CEBAS-CSIC; Murcia, Spain.

*Corresponding author. Email: janguita@cicbiogune.es; [hrodriguez@cicbiogune.es](mailto:hrodriguez@cicbiogune.es)

## MATERIALS AND METHODS

**Growth kinetics of *B. burgdorferi* in the presence of phloroglucinol**

*B. burgdorferi* were grown at 34 ºC in Barbour-Stoenner-Kelly (BSK)-H medium (Sigma- Aldrich) in 5 ml polystyrene round-bottom tubes in the presence or absence of phloroglucinol and counted in a dark field microscope (Zeiss, Munich, Germany).

**Phagocytosis assays for *B. burgdorferi***

Phagocytosis was assessed as previously described (1). BMMs were pretreated with 1mM phloroglucinol 1 h before the addition of GFP-expressing *B. burgdorferi* at an m.o.i. of 25. The cells were preincubated at 4 ºC prior to the addition of *B. burgdorferi* and immediately incubated at 37 ºC for 3 h. A replica plate was maintained at 4 ºC for the duration of the assay as a washing control. The cells were analyzed in a BD FACS Canto II (BD Biosciences, San Agustín de Guadalix, Spain). FlowJo version 10 was used for the analysis. The phagocytic index was calculated using the formula: % GFP cells (Test) × mean fluorescence intensity (MFI) (Test) − % GFP cells (4 ºC control) × MFI (4˚C control) (1).

# REFERENCES

1. Barriales D, Martin-Ruiz I, Carreras-Gonzalez A, Montesinos-Robledo M, Azkargorta M, Iloro I, et al. *Borrelia burgdorferi* infection induces long-term memory-like responses in macrophages with tissue-wide consequences in the heart. PLoS Biol. 2021;19(1):e3001062.


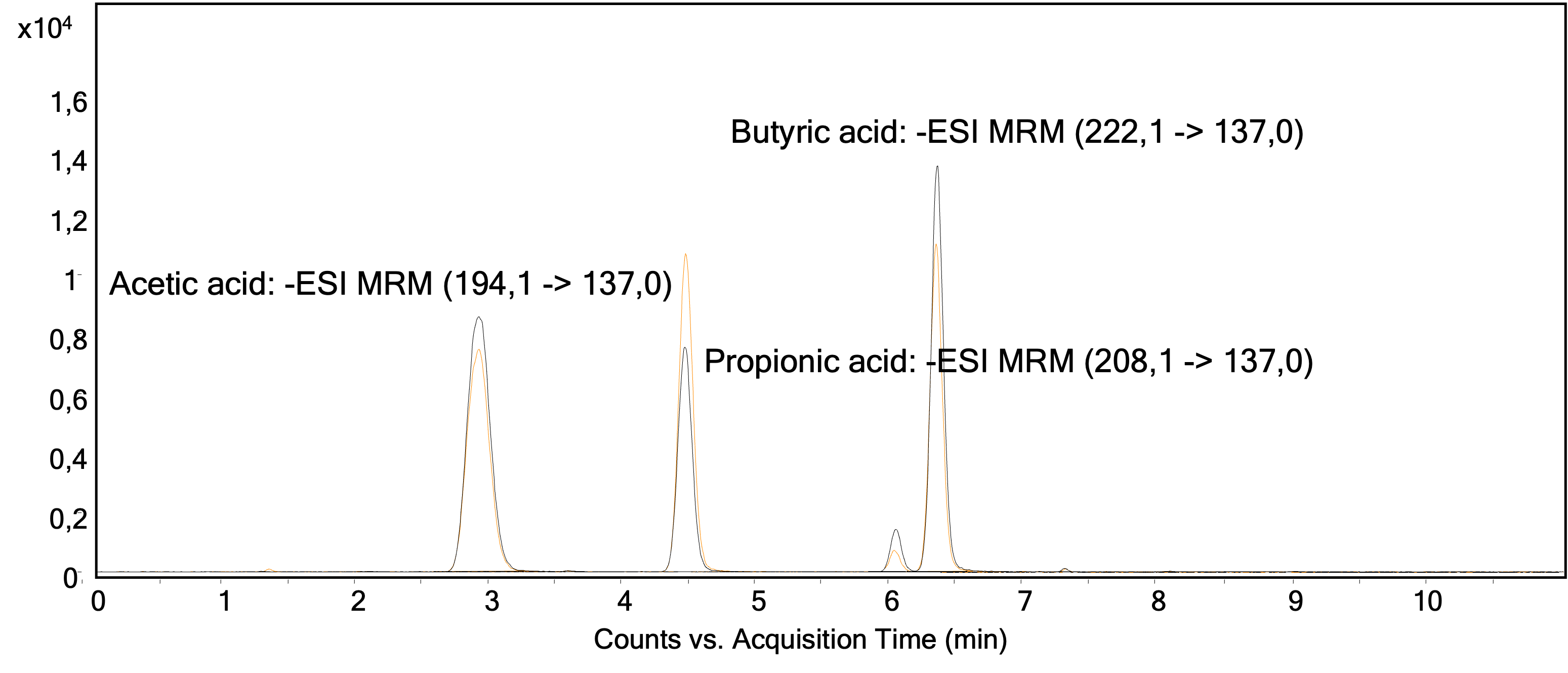


**Fig. S1. Extracted ion chromatograms of 3NPH derivatized SCFAs in fecal samples from two randomly selected mice (one control and one PG-treated) from LC-MS/MS analysis.** The chromatograms were obtained from the MRM transitions of acetic acid (m/z 194 → 137), propionic acid (m/z 208.1 → 137), and butyric acid (m/z 221.1 →

137). The black line represents the chromatogram of the control sample, while the orange line corresponds to the fecal sample from a PG-treated mouse.

A B C

2.5


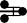

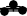

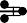

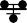


10^7^ Spirochetes/ml

2.0

1.5

1.0

0.5

0.0

*


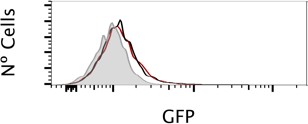


Ctrl 0.1 1 10

PG (mM)

8000

6000

Phagocytic index

4000

2000

0

ns

Ctrl PG

## Fig. S2. Phloroglucinol does not affect *B. burgdorferi* viability or phagocytosis by BMMs.

(**A**) Inhibitory effect of different concentrations of phloroglucinol on the growth of *B. burgdorferi*, measured after 5 days of incubation. Control spirochetes were cultured with 5% EtOH (Ctrl). All conditions were assessed in triplicate starting from the same initial inoculum. Statistical analysis was performed by one-way ANOVA, * p < 0.05. (**B**) Representative histogram of BMM phagocytosis of GFP-expressing *B. burgdorferi*. The cells were pretreated (red) with 1 mM phloroglucinol one h prior to their exposure to the spirochete. Black line: control. The grey histogram represents the 4 ºC control. (**C**) Phagocytic index of BMMs pretreated with phloroglucinol (orange) and controls (black). Phagocytosis experiments were performed with BMMs from 4 mice and represent 2 independent experiments.

✱

✱ ✱

Ctrl PG DSS

PG-DSS

✱

Mean Abundance ± SE

0.8

0.6

0.4

0.2

0.0

**Fig. S3. Relative abundance of microbiota phyla in phloroglucinol-treated, DSS-induced mice.** Histogram showing the mean abundance ± standard error of representative phyla after phloroglucinol treatment (d0, PG and controls -Ctrl) and after Phloroglucinol treatment followed by DSS induction (d8, PG-DSS and DSS).

***Camk1d Hexb Btg1***


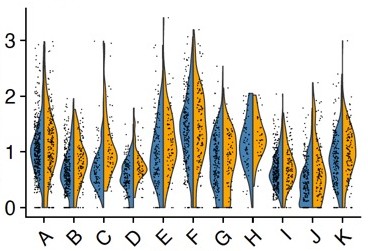


*


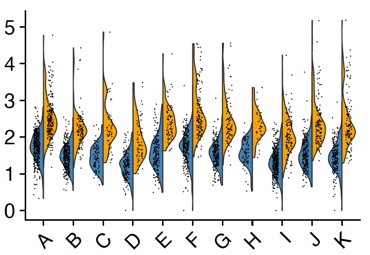


* * * * * * * * * * *


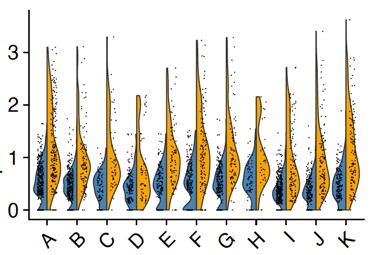


* * * * * * * * * *

***Gphn Lars2 Sub1***


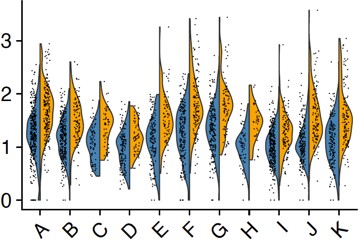


* * * * * * * * * *


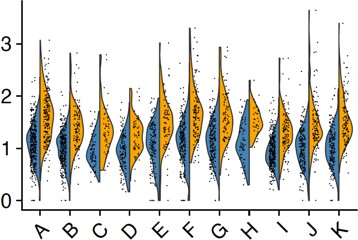


* * * * * * * * * * *


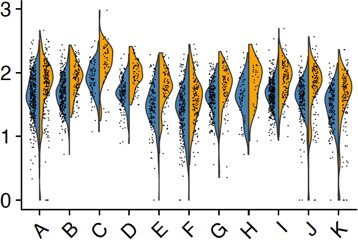


* * * * * * *

***Snhg9***


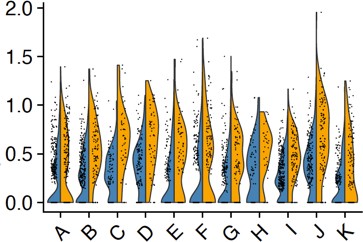


* * *

* * *

**Expression levels**


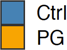


## Cluster Identity

**Fig. S4. Violin plots showing the expression levels in individual clusters of the genes regulated by the transcription factors PU.1-IRF, GFI1B and SP1.** Blue represents control (Ctrl) cells while phloroglucinol (PG)-treated cells are in orange. The asterisk (*) depicts those clusters with significantly different expression levels (FDR < 0.05). Cluster identification corresponds to those in Figure 5B.

**Table S1. Bacterial strains and culture conditions.**

| **Bacterial strain** | **Source** | **Medium**  **Culture conditions** |
| --- | --- | --- |
| *Borrelia burgdorferi*, strain B31,  clone 5A15 | Purser and Norris,  2000 | H-BSK  34 ºC, microaerophilic |
| *B. burgdorferi,* strain 297, clone  Bb914 (GFP+) | Dunham-Ems et al.,  2009 | H-BSK  34 ºC, microaerophilic |
| *Fusobacterium nucleatum* subsp.  *nucleatum* Knorr | ATCC 23726 | TSB + 0.25% L-Cys + 10% FBS  37 ºC, anaerobic |
| *Escherichia coli* (clinical isolate) | Hospital de Valdecilla | LB  37 ºC, agitation, aerobic |
| *Escherichia coli* Nissle 1917 (T7);  EcNT7 | DSM 115365 | LB  37 ºC, agitation, aerobic |
| *Akkermansia muciniphila* Muc | ATCC BAA-835 | BHI  37 ºC, anaerobic |
| *Prevotella copri* | DSM 18205 | GAM + 0.05% L-Cys  37 ºC, anaerobic |

**Table S2. Primers used.**

| **Gene** | **Species** | **Sequence** |
| --- | --- | --- |
| *Rpl19* Fwd | Mouse | 5’-GACCAAGGAAGCACGAAAGC-3’ |
| Rev |  | 5’-CAGGCCGCTATGTACAGACA-3’ |
| *Tnf* Fwd | Mouse | 5’-AGCCCACGTCGTAGCAAACCAC-3’ |
| Rev |  | 5’-ATCGGCTGGCACCACTAGTTGGT-3’ |
| *Adgre1* Fwd | Mouse | 5’-CTGCGCAGATGTTGATGAGTGTC-3’ |
| Rev |  | 5’-GGAGCCATTCAAGACAAAGCCTG-3’ |
| *RecA* Fwd | *B. burgdorferi* | 5’-GTGGATCTATTGTATTAGATGAGGCT-3’ |
| Rev |  | 5’-GCCAAAGTTCTGCAACATTAACACCT-3’ |
